# Engineering, Structure and Immunogenicity of the Human Metapneumovirus F Protein in the Postfusion Conformation

**DOI:** 10.1371/journal.ppat.1005859

**Published:** 2016-09-09

**Authors:** Vicente Más, Laura Rodriguez, Eduardo Olmedillas, Olga Cano, Concepción Palomo, María C. Terrón, Daniel Luque, José A. Melero, Jason S. McLellan

**Affiliations:** 1 Unidad de Biología Viral, Centro Nacional de Microbiología and CIBER de Enfermedades Respiratorias, Instituto de Salud Carlos III, Majadahonda, Madrid, Spain; 2 Unidad de Microscopía Electrónica y Confocal, Centro Nacional de Microbiología, Instituto de Salud Carlos III, Majadahonda, Madrid, Spain; 3 Department of Biochemistry and Cell Biology, Geisel School of Medicine at Dartmouth, Hanover, New Hampshire, United States of America; Georgia State University, UNITED STATES

## Abstract

Human metapneumovirus (hMPV) is a paramyxovirus that is a common cause of bronchiolitis and pneumonia in children less than five years of age. The hMPV fusion (F) glycoprotein is the primary target of neutralizing antibodies and is thus a critical vaccine antigen. To facilitate structure-based vaccine design, we stabilized the ectodomain of the hMPV F protein in the postfusion conformation and determined its structure to a resolution of 3.3 Å by X-ray crystallography. The structure resembles an elongated cone and is very similar to the postfusion F protein from the related human respiratory syncytial virus (hRSV). In contrast, significant differences were apparent with the postfusion F proteins from other paramyxoviruses, such as human parainfluenza type 3 (hPIV3) and Newcastle disease virus (NDV). The high similarity of hMPV and hRSV postfusion F in two antigenic sites targeted by neutralizing antibodies prompted us to test for antibody cross-reactivity. The widely used monoclonal antibody 101F, which binds to antigenic site IV of hRSV F, was found to cross-react with hMPV postfusion F and neutralize both hRSV and hMPV. Despite the cross-reactivity of 101F and the reported cross-reactivity of two other antibodies, 54G10 and MPE8, we found no detectable cross-reactivity in the polyclonal antibody responses raised in mice against the postfusion forms of either hMPV or hRSV F. The postfusion-stabilized hMPV F protein did, however, elicit high titers of hMPV-neutralizing activity, suggesting that it could serve as an effective subunit vaccine. Structural insights from these studies should be useful for designing novel immunogens able to induce wider cross-reactive antibody responses.

## Author Summary

Human metapneumovirus (hMPV) is a frequent cause of severe lower respiratory tract infections in very young children and a vaccine is not yet available. Protection against hMPV infection is afforded mainly by neutralizing antibodies directed against the fusion (F) glycoprotein. After iterative rounds of protein engineering, we generated a soluble form of the hMPV F protein in the postfusion conformation and determined its crystal structure. The structure is similar to that of the related human respiratory syncytial virus (hRSV) F glycoprotein, and two neutralizing epitopes are particularly well conserved, thus providing a structural basis for the cross-neutralizing activity of several monoclonal antibodies. Immunization of mice with the engineered hMPV F postfusion protein elicited high hMPV-neutralizing antibody titers, suggesting that this protein could be an attractive subunit vaccine antigen. These results also open the possibility of designing novel cross-protective immunogens.

## Introduction

Human metapneumovirus (hMPV) was first isolated in 2001 from respiratory specimens collected from children with respiratory tract infections [1]. Sequence analysis was used to classify hMPV in the *Metapneumovirus* genus of the Pneumovirinae subfamily of paramyxoviruses. This subfamily also includes the *Pneumovirus* genus in which human respiratory syncytial virus (hRSV) is the best known prototype. Like all members of the Paramyxovirus family, hMPV and hRSV are enveloped, single-stranded, negative-sense RNA viruses that share many characteristics of their respective life cycles with other paramyxoviruses [2]. Sequence analysis of hMPV samples indicate that there are two main genetic lineages (A and B), each divided into at least two sub-lineages (A1, A2, B1 and B2) [3].

Clinical manifestations of hMPV infections are similar to those of hRSV, ranging from mild respiratory illness to bronchiolitis and pneumonia in children less than five years of age [2]. Although the frequency of severe lower respiratory tract infections is highest for hRSV, hMPV contributes to a significant fraction of the worldwide burden of bronchiolitis and pneumonia in young children [4]. As for hRSV, hMPV infections are also a frequent cause of morbidity and mortality in the elderly [5,6] and immunocompromised adults [7,8]. Despite their clinical significance, vaccines are not yet available for hMPV and hRSV.

hMPV encodes three glycoproteins (SH, G and F) that are inserted into the viral membrane. The SH protein is a small hydrophobic protein whose function is unknown, although it has been claimed to inhibit NF-kappaB transcriptional activity [9]. The G glycoprotein is heavily glycosylated with multiple *O*- and *N*-linked sugar chains, resembling mucins [10], and serves as the putative viral attachment protein via interactions with cell-surface factors such as proteoglycans [11]. Finally, the fusion (F) glycoprotein mediates fusion of the viral and cellular membranes to allow entry of the viral ribonucleoprotein into the cell cytoplasm and thus initiate a new infectious cycle [12,13].

Recombinant hMPV with deletion of the G gene, the SH gene or both, retains the ability to replicate in epithelial cell lines, although these viruses have an attenuated phenotype *in vivo* [14]. Hence, at least in the G-deletion mutants, the F glycoprotein has to perform both the attachment and fusion steps. Indeed, it has been shown that F can bind to cell-surface molecules, such as proteoglycans [15] and certain integrins [16]. The interaction of the F glycoprotein with integrins requires a RGD motif conserved in all hMPV strains [12,17], and the interaction likely occurs after the initial binding of hMPV F to proteoglycans [15].

Paramyxovirus membrane fusion is thought to occur at the plasma membrane in a pH-independent manner. However, it was recently shown that hMPV particles are internalized via clathrin-mediated endocytosis in a dynamin-dependent manner [18] before pH-independent fusion of the viral and endosomal membranes takes place, except for a minority of strains that require acidic pH for efficient membrane fusion [19,20]. Even in certain cells, such as monocyte-derived dendritic cells, hMPV uptake occurs preferentially by macropinocytosis, a process that is partially inhibited by SH and G glycoproteins [21]. In all cases, however, hMPV F is the main player in the membrane fusion process.

hMPV F is a class I fusion glycoprotein, synthesized as an inactive precursor (F0) that needs to be cleaved to become fusion competent. Proteolytic cleavage generates two disulfide-linked subunits (F2 N-terminal to F1) that assemble into a homotrimer. Cleavage occurs at a monobasic cleavage site immediately upstream of the hydrophobic fusion peptide. Cleavage can be achieved in tissue culture by addition of trypsin to the medium [19,22] but *in vivo* other serine proteases, such as TMPRSS2, are likely to be more relevant for cleavage [23].

The F trimer is incorporated into the virus particle in a metastable, “prefusion” conformation. To initiate membrane fusion, hMPV F is activated by still ill-defined mechanisms leading to a series of stepwise conformational changes in the F protein that drive membrane fusion and result in hMPV F adopting a highly stable “postfusion” conformation. Much of our current knowledge about the F protein conformational transition comes from the atomic structures of related paramyxovirus F proteins in either the prefusion [24–27] or postfusion conformation [28–31]. Among other changes, the prefusion-to-postfusion transition includes formation of a pre-hairpin intermediate in which heptad repeat A (HRA) sequences of the F1 subunit refold into a long continuous α-helix. This allows insertion of the hydrophobic fusion peptide, located at the N-terminus of HRA, into the target membrane. Refolding of this intermediate leads to merging of the viral and target membranes concurrently with the assembly of HRA and HRB sequences of the F1 subunit into a highly stable six-helix bundle (6HB), which is characteristic of the postfusion conformation [32].

Protection against hMPV infection is mediated mainly by neutralizing antibodies that presumably block refolding of the F glycoprotein and hence membrane fusion [33]. In contrast to other paramyxoviruses, the F glycoprotein is the only viral antigen of hMPV capable of inducing neutralizing and protective antibodies [34]. In addition, antibodies to the G glycoprotein are not protective [35]. Escape mutants selected with hMPV F-specific monoclonal antibodies (mAbs) have identified residues located in the hMPV F protein primary structure at sites equivalent to those of the antigenic sites identified in hRSV F [36]. Recently, mAb 54G10, isolated against hMPV F, was shown to cross-neutralize hRSV *in vitro* and to protect BALB/c mice against hRSV infection [37]. Another mAb, MPE8, was also recently described that cross-neutralized not only hMPV and hRSV but additionally two other viruses of the Pneumovirinae subfamily: bovine RSV and pneumonia virus of mice [38]. However, a global picture of the cross-reactivity potential of hMPV and hRSV F-specific antibodies is still missing.

In order to advance our understanding of hMPV F structure and antigenicity (which lags behind other paramyxoviruses), we engineered a homogeneous preparation of soluble hMPV F folded in its postfusion conformation. This process required genetic manipulations that were not necessary for other paramyxovirus F proteins. We also crystallized the stabilized hMPV F postfusion trimer and determined its structure by X-ray diffraction analysis. Comparison with the postfusion hRSV F structure revealed a high degree of similarity between the two proteins in multiple regions, including a previously characterized antigenic site of hRSV recognized by mAb 101F [39,40]. Indeed, we found that 101F binds to hMPV F and cross-neutralizes hMPV. Although immunization of BALB/c mice with purified postfusion hMPV F induced very limited cross-binding and cross-neutralization with hRSV, the elicited sera had robust neutralizing activity against hMPV, indicating that the hMPV postfusion F protein may be an effective subunit vaccine antigen.

## Materials and Methods

### Ethics statement

Animal studies were performed under the regulations of the Spanish and European legislation concerning vivisection and the use of genetically modified organisms. Protocols were approved by the “Comité de Ética de la Investigación y del Bienestar Animal” of “Instituto de Salud Carlos III” (CBA PA 19_2012).

### Expression and purification of soluble hMPV postfusion F glycoprotein

**Fig 1:**
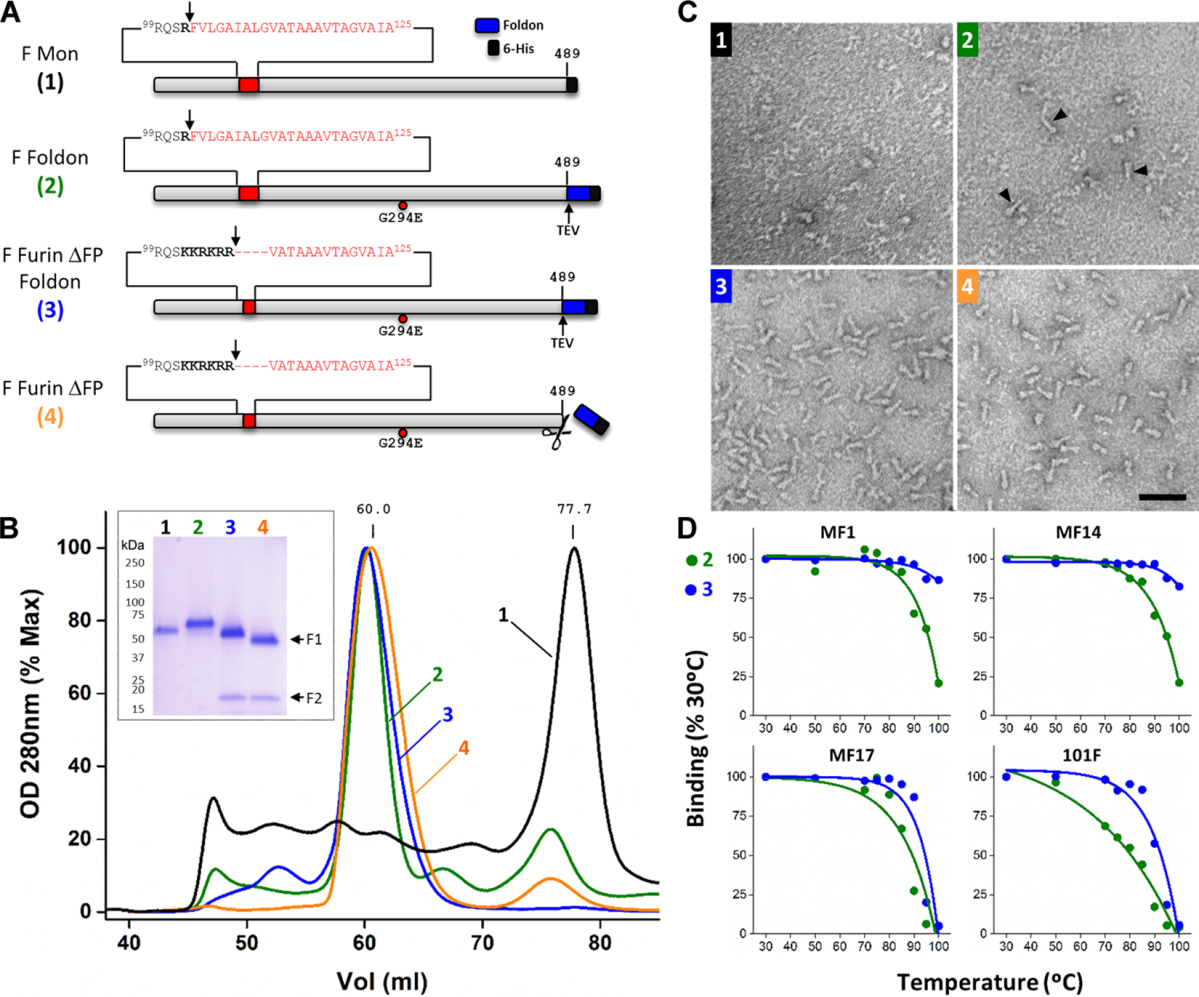
Stabilization and characterization of the soluble hMPV F protein ectodomain as a postfusion trimer. **A**) Diagrams of the different constructs of hMPV F used in this study. (1) Scheme of the F protein (grey rectangle, NL/1/00 strain) denoting the last amino acid of its ectodomain (489), the fusion peptide (red), the preceding cleavage site (arrow) and the C-terminal 6xHis-tag (black box). (2) Same scheme but with the Foldon sequence (blue rectangle) and TEV cleavage site (arrow) added, and the change G294E denoted by a red circle. (3) The basic residues of hRSV F cleavage site II are shown in boldface and the amino acids deleted from the fusion peptide are indicated by dashes in the amino acid sequence. (4) Scheme of the protein after TEV cleavage. **B**) Gel-filtration traces of the four proteins depicted in panel A, labelled and color-coded as in panel A. Inset shows a Coomassie-blue-stained SDS-PAGE, run under reducing conditions, of the major peak of each chromatogram. **C**) Electron microscopy of negative-stained proteins 1–4. Some cone-shaped molecules are indicated in panel 2 by black arrowheads. Scale bar: 50 nm. **D**) Proteins 2 (green) and 3 (blue) were heated stepwise at the indicated temperatures, as described in Materials and Methods, before being tested for binding in ELISA to the mAbs indicated in each panel. Results are shown as percent of binding with proteins heated for 10 minutes at 30°C.

The F protein ectodomain (amino acids 1–489, see Fig 1) was amplified from a pCAGG plasmid carrying the F gene from either the NL/1/00 strain (A1 sublineage) or the NL/1/99 strain (B1 sublineage) of hMPV [41]. Amplification was carried out with forward and reverse primers containing sequences from the beginning and the end of the F ectodomain and a C-terminal 6xHis-tag, incorporating EcoRI and NcoI sites. After digestion with these enzymes, the amplified DNA was inserted in the pRB21 plasmid digested with the same enzymes (Fig 1A, F Mon, protein 1). Subsequently: i) the amino acid change G294E was introduced in the F protein of the A1 sublineage since B1 sublineage already has Glu at position 294 and ii) the foldon trimerization domain [42] was added at the C-terminus of the F protein ectodomain, flanked upstream by a TEV protease site and downstream by a Xa protease site, followed by the 6xHis-tag (Fig 1A, F Foldon, protein 2). Hence, the complete amino acid sequence of the C-terminal appendage was: *SGRENLYFQG*GGG**GSGYIPEAPRDQAYVRKDGEWVLLSTFL**GG*TEGR*HHHHHH. TEV and Xa sequences are in italics and underlined and foldon sequences are in boldface. Other sequences correspond to linkers and histidines.

pRB21 plasmid encoding the F protein of Fig 1A was further mutagenized by PCR, using the Phusion Site-Directed Mutagenesis kit (ThermoFisher Scientific) to: i) insert sequences of cleavage site II from hRSV F into the hMPV F cleavage site and ii) delete the first nine residues of the fusion peptide (amino acids 103–111, F Furin ΔFP Foldon, protein 3). The different recombinant plasmids were used to generate the matching recombinant vaccinia viruses by the method of Blasco and Moss [43].

CV-1 cells were infected with vaccinia viruses expressing the hMPV F protein ectodomains (moi 0.05 pfu/cell) of the previous paragraphs (Fig 1A). In the case of constructs 3 and 4 shown in Fig 1A, cells were additionally co-infected with a vaccinia virus recombinant that expresses furin (moi 0.03 pfu/cell) (a kind gift of Manuel Ramos, Centro Nacional de Microbiología, Madrid) [44]. In all cases, culture supernatants were collected 48 hours after infection, and these were concentrated and buffer-exchanged using Vivaflow membranes (Sartorius). Then, they were loaded onto Ni^2+^ columns in 50 mM Na_2_HPO_4_ pH 8.0, 300 mM NaCl, 10 mM imidazole buffer and, after washing, proteins were eluted with the same buffer containing 250 mM imidazole. Finally, proteins were concentrated with Amicon (Millipore) and exchanged to buffer without imidazole before being loaded onto a HiLoad 16/600 Superdex 200 pg gel filtration column (GE Healthcare) equilibrated and eluted with the same buffer (Fig 1B).

Protein purity and integrity were checked by SDS-PAGE and Coomassie-blue staining under reducing conditions. Thermostability of purified proteins was assessed by heating samples in different tubes in a thermoblock. Starting at 30°C, samples were incubated for 10 minutes at this temperature before raising it to the next step and incubation continued for another 10 minutes. This stepwise increase in temperature and incubation was repeated until reaching 100°C. After incubation at each temperature, one of the sample tubes of each protein was withdrawn and kept at 4°C until the end of the incubation period. Then proteins were tested for antibody binding in ELISA as indicated below.

### Electron microscopy

Purified proteins were applied to glow-discharged carbon-coated grids and negatively stained with 1% aqueous uranyl formate. Images were recorded with a Gatan ERLANGHEN 1000 W CCD camera in a JEOL JEM-1011 electron microscope operated at 100 kV at a detector magnification of 20,400X or a FEI Eagle CCD camera in a Tecnai G2 electron microscope operated at 200 kV at a detector magnification of 69,444X. Xmipp software package [45] was used to select 112 and 100 images of 101F Fab in complex with hRSV F and hMPV F, respectively, and to obtain 2D averages with the CL2D routine.

### Crystallization and data collection

Crystallization conditions for Foldon-removed hMPV A1 postfusion F protein at 5 mg/ml in 2 mM Tris-HCl pH 8.0, 200 mM NaCl were screened by the sitting-drop vapor-diffusion method with an NT8 nanoliter-volume liquid handler (Formulatrix). Initial crystallization hits were obtained in 16.8% PEG 3,350, 10% 2-methyl-2,4-pentanediol (MPD), 0.2 M lithium sulfate and 0.1 M imidazole pH 6.5 [46]. The crystal used for structure determination was grown in 18.5% PEG 3,350, 11% MPD, 0.2 M lithium sulfate, 0.01 M nickel chloride and 0.1 M imidazole pH 7.0 at a 2:1 protein:reservoir ratio. Crystals were directly frozen in liquid nitrogen and data were collected to 3.3 Å resolution at the Structural Biology Center beamline 19-ID (Advanced Photon Source, Argonne National Laboratory).

### Structure determination

Diffraction data were processed using the CCP4 program suite; data were indexed and integrated in iMOSFLM [47] and scaled and merged with AIMLESS [48]. A molecular replacement solution was found by PHASER [49] using a search model generated by replacing domains in the hRSV F postfusion structure (PDB ID: 3RRR) [29] with those from the antibody-bound monomeric fragment of hMPV F (PDB ID: 4DAG) [50]. The structure was built manually in COOT [51] and refined with PHENIX [52] using non-crystallographic symmetry restraints. PHENIX-generated feature-enhanced maps were particularly helpful during this process [53]. Data collection and refinement statistics are presented in Table 1.

**Table 1:** Crystallographic data collection and refinement statistics.

	hMPV Postfusion F
**Data collection**	
Space group	*P*4_1_2_1_2
Cell constants	
*a*, *b*, *c* (Å)	128.7, 128.7, 572.8
α, β, γ (°)	90, 90, 90
Wavelength (Å)	0.9793
Resolution (Å)	37.0–3.3 (3.37–3.30)
*R*_merge_	0.216 (1.015)
*R*_pim_	0.099 (0.461)
*I* / σ*I*	5.2 (1.9)
CC(1/2)	0.993 (0.802)
Completeness (%)	99.8 (99.9)
Redundancy	5.4 (5.6)
**Refinement**	
Resolution (Å)	37.0–3.3 (3.34–3.30)
No. reflections	73,071 (2,709)
*R*_work_ / *R*_free_ (%)	22.1/27.0
No. atoms	
Protein	20242
Ion (SO4)	25
Glycan (NAG, FUC)	274
*B*-factors	
Protein	74.7
Ion (SO4)	116.4
Glycan (NAG, FUC)	110.9
R.m.s. deviations	
Bond lengths (Å)	0.008
Bond angles (°)	1.12
Ramachandran	
Favored (%)	97.6
Allowed (%)	2.4
Outliers (%)	0.0

### Surface plasmon resonance

All experiments were carried out on a Biacore X100 using single-cycle format. Anti-mouse IgG (GE Healthcare) was covalently coupled to both the sample and reference cells of a CM5 chip at 10,000 response units (RU). Approximately 200 RU of mAb 101F were bound to the anti-mouse IgG. Then, purified proteins were injected at five different concentrations, as noted in the figure legends, at a flow rate of 50 μl/min. Association and dissociation phases were 108 seconds and 300 seconds, respectively. The chip was regenerated using 30 mM HCl, and the binding data were fit to a 1:1 Langmuir binding model for the calculation of the kinetic parameters *k*_on_ and *k*_off_. The *K*_D_ was then calculated as the ratio of these two rate constants (*k*_off_/*k*_on_).

### Enzyme-linked immunosorbent assay (ELISA)

Four hundred nanograms of the mAbs indicated in the figure legends was used to coat each well of 96-well microtiter plates for 16 hours at 4°C. Non-specific binding was blocked with 0.5% bovine serum albumin (BSA) in PBS. Then, serial dilutions of soluble proteins were added and incubated for 1 hour at room temperature, followed by an excess of a biotinylated anti-His mAb, streptavidin-peroxidase and OPD (Sigma) substrate. Extensive washing with water was done after each step. Optical density was measured at 490 nm.

In the case of mouse sera, the 96-well plates were coated with 40 ng/well of purified protein in PBS. Non-specific binding was blocked as described above. Then, serial dilutions of sera in blocking solution were added, and bound antibodies were detected with peroxidase-labelled goat anti-mouse Igs and OPD as substrate (GE Healthcare).

### Microneutralization test

Predetermined amounts of GFP-expressing hMPV recombinant viruses (NL/1/00 A1 sublineage or NL/1/99 B1 sublineage, a kind gift of Bernadette van den Hoogen and Ron Fouchier, Rotterdam, the Netherlands) or GFP-hRSV (A2 strain, a kind gift of Mark Peeples, Columbus, Ohio, USA) were mixed with serial dilutions of mouse serum before being added to cultures of Vero-118 cells. Twenty-four to forty-eight hours later, the medium was removed, PBS was added and the amount of GFP per well was measured with a Tecan microplate reader M200. Fluorescence values were represented as percent of a virus control without antibody.

### Immunostaining and flow cytometry

Vero-118 cells growing in either 8-well chamber slides or 24-well plates were infected with hMPV_A1_-GFP virus for 36 hours. At this time, medium was removed and after washing with PBS, cells were incubated sequentially with the mAbs of interest (30 ng/μl) followed by biotinylated sheep anti-mouse or anti-human IgG (GE Healthcare) and then streptavidin-RPE (red phycoerythrin, Southern Biotech). After washing with PBS, cells in the chamber slides were fixed with 2% paraformaldehyde in PBS and observed with a Leica TCS SP5 AOBS confocal microscope whereas cells in the 24-well plates were detached with 5 mM EDTA in PBS, fixed with 1% paraformaldehyde and analyzed with a Becton Dickinson FACSCanto analyzer.

### Immunization of BALB/c mice with purified soluble hMPV F and hRSV F ectodomain

Groups of five BALB/c female mice (8 weeks old, from Envigo Rms Spain) were inoculated once intramuscularly in the hind legs with 10 μg of the soluble postfusion hMPV F A1 strain that was used for crystallization or the equivalent protein of the B1 strain. In addition, mice were inoculated with soluble postfusion hRSV F expressed similarly to the hMPV proteins [54]. Samples in 50 μl of PBS were mixed with an equal volume of CpG. Four weeks later, mice were euthanized, blood was collected and sera were obtained after coagulation.

## Results

### Engineering a soluble form of hMPV F stabilized in the postfusion conformation

Following strategies previously used for the expression of postfusion F protein ectodomains from hRSV [29,54,55], parainfluenza virus type 3 (hPIV3) [31] and Newcastle disease virus (NDV) [30], sequences encoding amino acids 1–489 of the hMPV F protein ectodomain with a C-terminal 6xHis-tag were inserted into a vaccinia virus recombinant by the method of Blasco and Moss [43] (Fig 1A, construct 1). CV-1 cells infected with this vaccinia virus produced a soluble F protein that was secreted into the culture medium since it lacked the transmembrane (TM) region and the cytoplasmic tail. Whereas the soluble ectodomains of hRSV, hPIV3 and NDV F proteins could be purified as postfusion trimers, hMPV F_TM_- eluted from the gel-filtration column at a retention volume corresponding to a monomer (Fig 1B, black line) and migrated as an uncleaved band of the expected size in SDS-PAGE (inset). Negatively stained samples of this hMPV F_TM_- protein did not show discernable macromolecular assemblies when observed by electron microscopy (EM) (Fig 1C, panel 1).

Addition of the fibritin trimerization domain (Foldon) from T4 bacteriophage [42] to the C-terminus of the hMPV F ectodomain (Fig 1A_,_ construct 2) shifted elution of the new F_TM_- protein towards the size of a trimer (Fig 1B, green line). The amino acid change G294E, which is found in other hMPV strains [41] was introduced in this construct after observing that this change increased protein expression. Although the protein was heterogeneous when observed by EM, some cone-shaped molecules that resembled the previously described postfusion hRSV F trimer became visible [55] (Fig 1C, panel 2). The source of this heterogeneity has not been investigated further.

hMPV growth in cell culture requires addition of trypsin to the culture medium to cleave the F protein at the monobasic site preceding the fusion peptide. Since no trypsin was present during production and purification of the hMPV F_TM_- monomer or trimer, these proteins remained uncleaved after purification, as seen by SDS-PAGE (Fig 1B, inset). Because circumstantial evidence suggests that cleavage enhances stability of paramyxovirus postfusion F proteins, the uncleaved hMPV F postfusion trimer of Fig 1A (construct 2) was treated with limited amounts of trypsin. Essentially all trypsin-treated molecules were seen by EM as cone-shaped molecules aggregated in rosettes (S1 Fig), presumably by intermolecular interactions of their respective fusion peptides, as previously reported for hRSV F [56].

To promote cleavage of the soluble hMPV F trimer without added trypsin, the hMPV F cleavage site was replaced with the second furin-cleavage site of hRSV F. In addition, to prevent protein aggregation, the first nine amino acids of the fusion peptide (residues 103–111) were deleted. The modified hMPV F protein eluted from the gel-filtration column as a partially cleaved trimer. To increase cleavage efficiency, cells were co-infected with a vaccinia virus expressing recombinant furin [44]. The soluble hMPV F protein now eluted as a trimer of fully cleaved protomers, as seen by SDS-PAGE (Fig 1B, inset), and existed as a homogeneous population of cone-shaped molecules, as observed by EM (Fig 1C, panel 3).

Thermostability of the uncleaved and cleaved F molecules (Fig 1A, constructs 2 and 3) was assessed by testing their reactivity with four different mAbs (MF1, MF14, MF17 and 101F) after heating stepwise up to 100°C (Fig 1D). mAb MF1 recognizes the 6HB domain of postfusion F [54], whereas mAbs MF14 and MF17 recognize neutralizing, non-overlapping epitopes that have not yet been mapped on hMPV F. Lastly, 101F is a site IV-specific mAb raised against hRSV F which cross-reacts with hMPV F (described in more detail below). Binding of mAbs MF1 and MF14 was essentially unchanged after heating the cleaved hMPV F protein up to 100°C, whereas binding was lost to great extent with uncleaved F at that temperature. Although reactivity of mAbs MF17 and 101F with the two proteins was lost after heating at 100°C, that loss occurred at temperatures 5–10°C lower with the uncleaved protein. Therefore, these results lend support to the idea that cleavage increases the stability of postfusion hMPV F and differ from those reported with hRSV F, in which changes in reactivity with three different mAbs after heating were essentially the same for uncleaved and cleaved postfusion F [57].

Finally, the F protein described in the previous paragraph was cleaved with TEV protease to release the Foldon domain and affinity tag (Fig 1A, construct 4), which were separated from the authentic hMPV F ectodomain by gel filtration (Fig 1B, orange line). Removal of these residues made the F1 subunit migrate faster in SDS-PAGE (Fig 1B, inset) and the cone-shaped molecules looked slightly shorter by EM (Fig 1C, panel 4), demonstrating that the proteolysis was complete.

### Structure of postfusion hMPV F and similarities with hRSV F

Crystals of the hMPV F A1 subtype were obtained in space group *P*4_1_2_1_2 and after optimization diffracted X-rays to 3.3 Å resolution. A molecular replacement solution was obtained using a composite search model containing regions from the postfusion hRSV F trimer structure [29] and the antibody-bound hMPV F monomer structure [50]. The asymmetric unit contained two postfusion hMPV F trimers, which allowed non-crystallographic symmetry restraints to be used during refinement. After manual building, the structure was refined to an *R*_work_ and *R*_free_ of 22.1% and 27.0%, respectively, with no Ramachandran outliers as determined by MolProbity [58,59]. Data collection and refinement statistics are presented in Table 1.

The structures of the two trimers in the asymmetric unit are very similar, with a root-mean-square deviation (rmsd) of 0.26 Å for 1,325 Cα atoms. The structures are nearly complete, with no missing loops, and only a few disordered residues at the C-terminus of the F2 subunit and at the N- and C-termini of the F1 subunit. In addition, electron density for one or more core glycans is visible at some of the three *N*-linked glycosylation sites (N57, N172 and N353) on the different polypeptide chains in the asymmetric unit. Since the protein used for crystallization and structure determination was expressed from CV-1 cells without the addition of any glycosylation inhibitors or treatment of the protein with endoglycosidases, complex-type glycans are likely present at each *N*-linked site, with the electron density for most of the glycans being disordered due to heterogeneity and flexibility.

**Fig 2:**
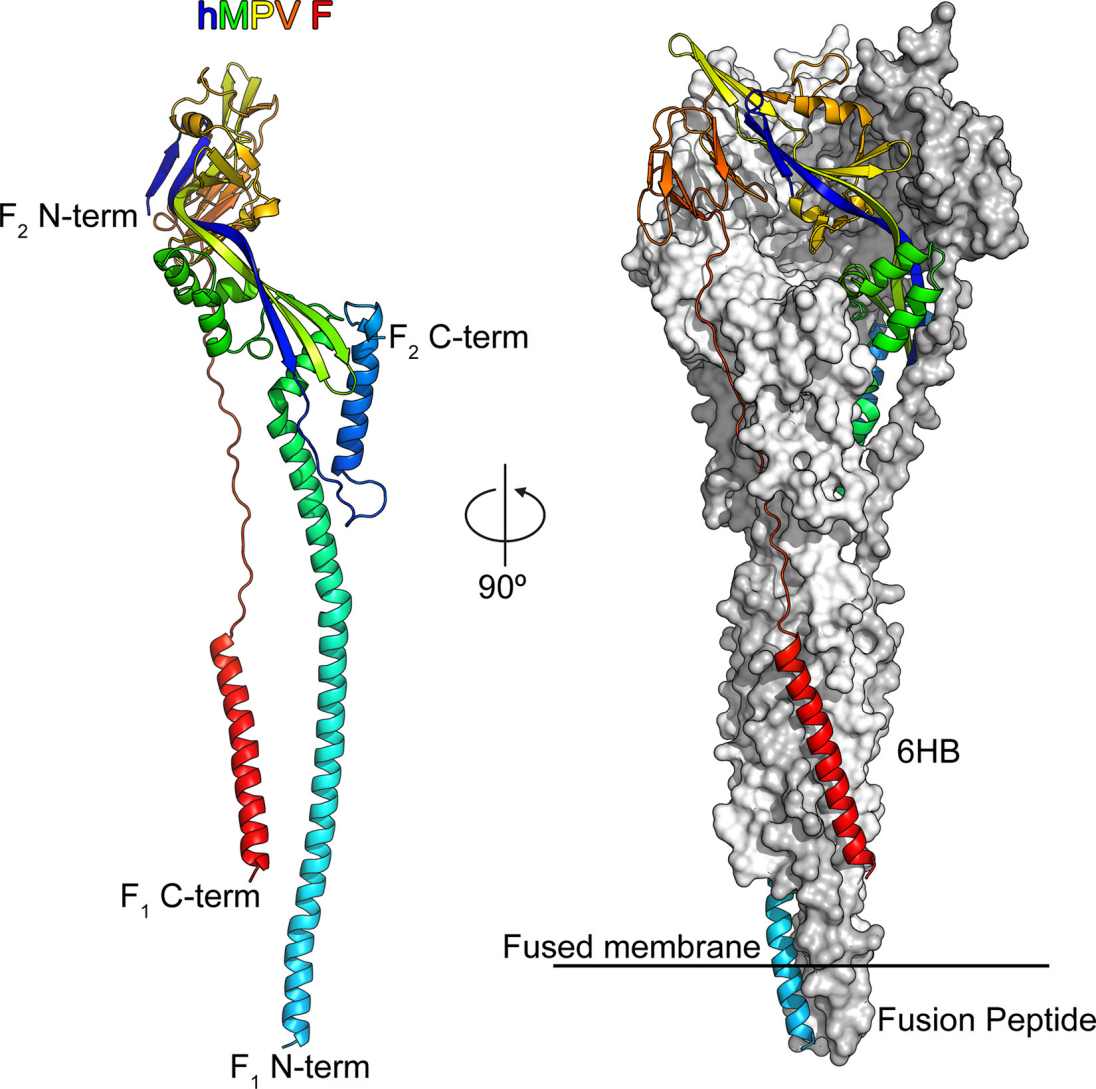
Structure of hMPV F in the postfusion conformation. *Left*: One protomer of the postfusion hMPV F trimer is shown as a ribbon colored as a rainbow from the N-terminus of F2 (blue) to the C-terminus of F1 (red). *Right*: The postfusion hMPV F trimer with one protomer shown as a ribbon and two protomers shown as molecular surfaces colored white and grey. The six-helix bundle (6HB) and fusion peptides are labeled.

**Fig 3:**
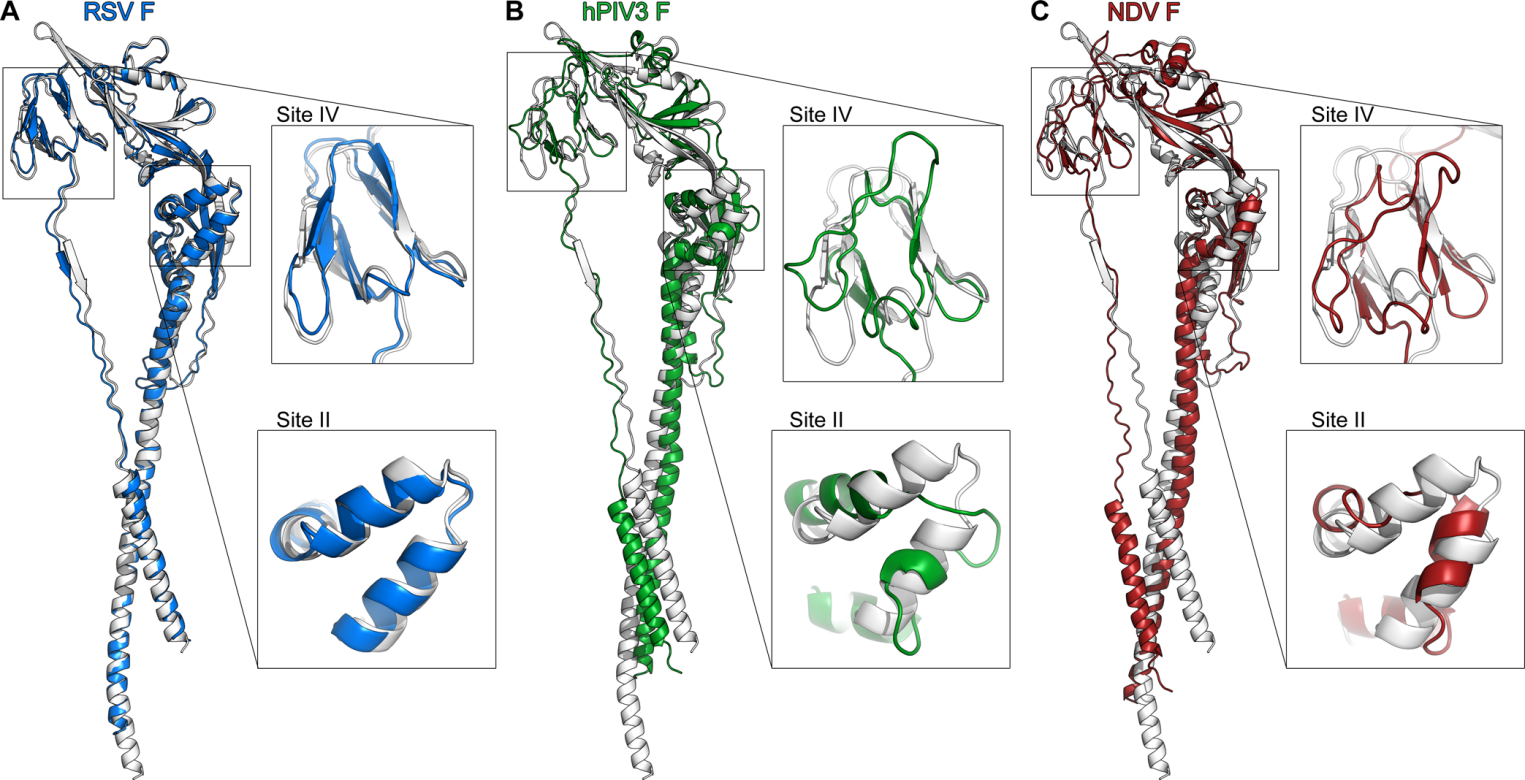
Comparison of paramyxovirus postfusion F structures. Superposition of one protomer of the postfusion hMPV F structure (white) with the corresponding structures of **A**) hRSV F, **B**) hPIV3 F, and **C**) NDV F. Two neutralizing antigenic sites (II and IV of hRSV F) are magnified.

The overall shape of the trimeric postfusion hMPV F protein resembled that of an elongated cone (Fig 2), consistent with the images observed by negative-stain EM (Fig 1C) and with previously determined structures of F proteins in the postfusion conformation from PIV3 [31], NDV [60] and hRSV [28,29]. The mature ectodomains of hMPV F and hRSV F have approximately 38% sequence identity, and overall their postfusion structures are similar, with an rmsd of 1.48 Å for 419 Cα atoms in an F2–F1 protomer (Fig 3A). Secondary structures are well conserved, as are the conformations of the two major neutralizing epitopes retained on the postfusion conformation (antigenic sites II and IV of hRSV F). In contrast, the hMPV postfusion F structure has a much greater divergence from the hPIV3 (Fig 3B) and NDV (Fig 3C) postfusion F structures. Although the overall folds are similar, the secondary structures do not align well, consistent with the lower sequence conservation between hMPV and these two Paramyxovirinae subfamily members. The poor conservation of antigenic sites II and IV suggest that it is unlikely that a single antibody against either of these sites could neutralize viruses in both paramyxovirus subfamilies.

### Binding and cross-neutralization of antibody 101F

101F is a widely used mAb, originally produced by a hybridoma obtained from mice inoculated with a recombinant vaccinia virus expressing the hRSV F glycoprotein from the Long strain. The 101F epitope has been mapped to antigenic site IV of hRSV F [39,61], and a crystal structure of 101F Fab bound to a peptide corresponding to a linear portion of the epitope has been determined [40]. This structure was used in modeling studies to identify additional contact residues in prefusion [25] and postfusion [29] hRSV F.

**Fig 4:**
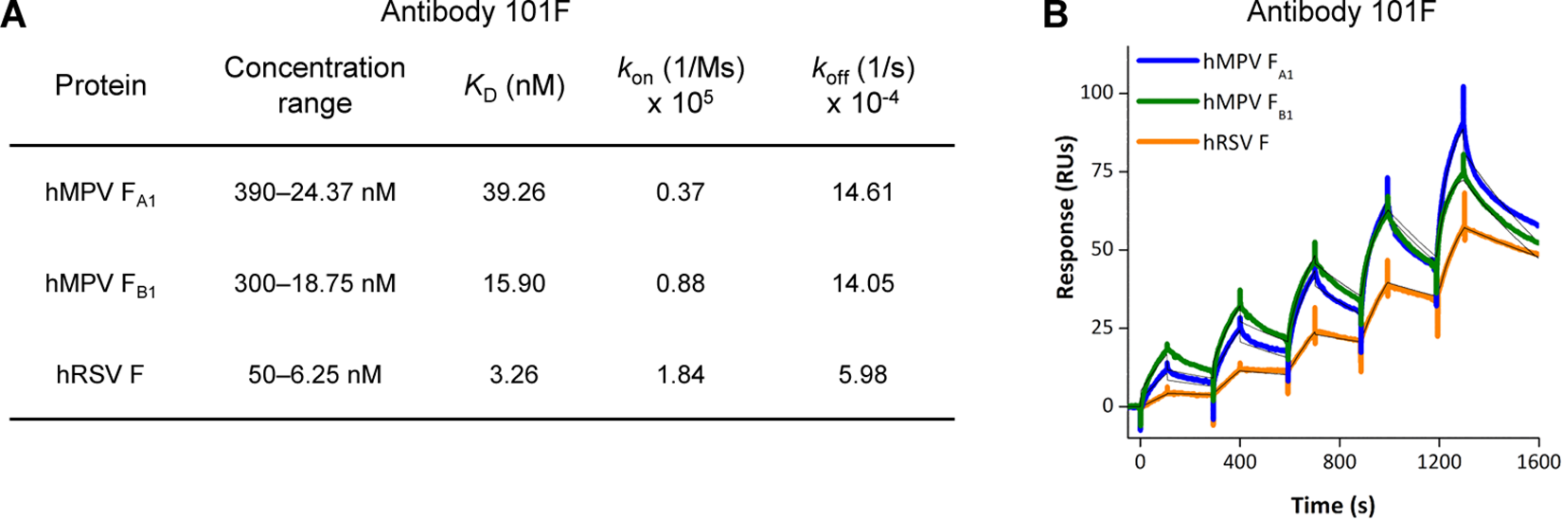
Binding of mAb 101F to hMPV and hRSV F proteins. **A**) Binding constants of mAb 101F for postfusion F proteins from hMPV NL/1/00 (A1 sublineage) and NL/1/99 (B1 sublineage) strains and from hRSV (Long strain) determined by surface plasmon resonance. **B**) Biacore binding sensorgrams used to determine the data in panel A.

Given the structural similarities of soluble postfusion trimers of hMPV and hRSV F, particularly in antigenic site IV (Fig 3A), binding of mAb 101F to both proteins was tested by surface plasmon resonance (SPR) (Fig 4). Two preparations of fully cleaved postfusion hMPV F were included in the assay: one derived from A1 sublineage (strain NL/1/00), whose structure is shown in Fig 2, and the other derived from B1 sublineage (strain NL/1/99), which was also observed as a population of homogeneous cones by EM (S2 Fig). In parallel, a previously described soluble form of postfusion hRSV F was also tested for binding to mAb 101F [55,62]. Association rate constants (*k*_on_) for the binding of 101F to both hMPV F proteins were about 2–5 times slower than that to hRSV F, and dissociation rate constants (*k*_off_) for 101F binding to the hMPV F proteins were almost three times faster than that to hRSV F. Consequently, the affinities (*K*_D_) of 101F for the soluble hMPV F proteins were 5–10 times weaker than for hRSV F (Fig 4A), but remained in the 15–40 nM range.

**Fig 5:**
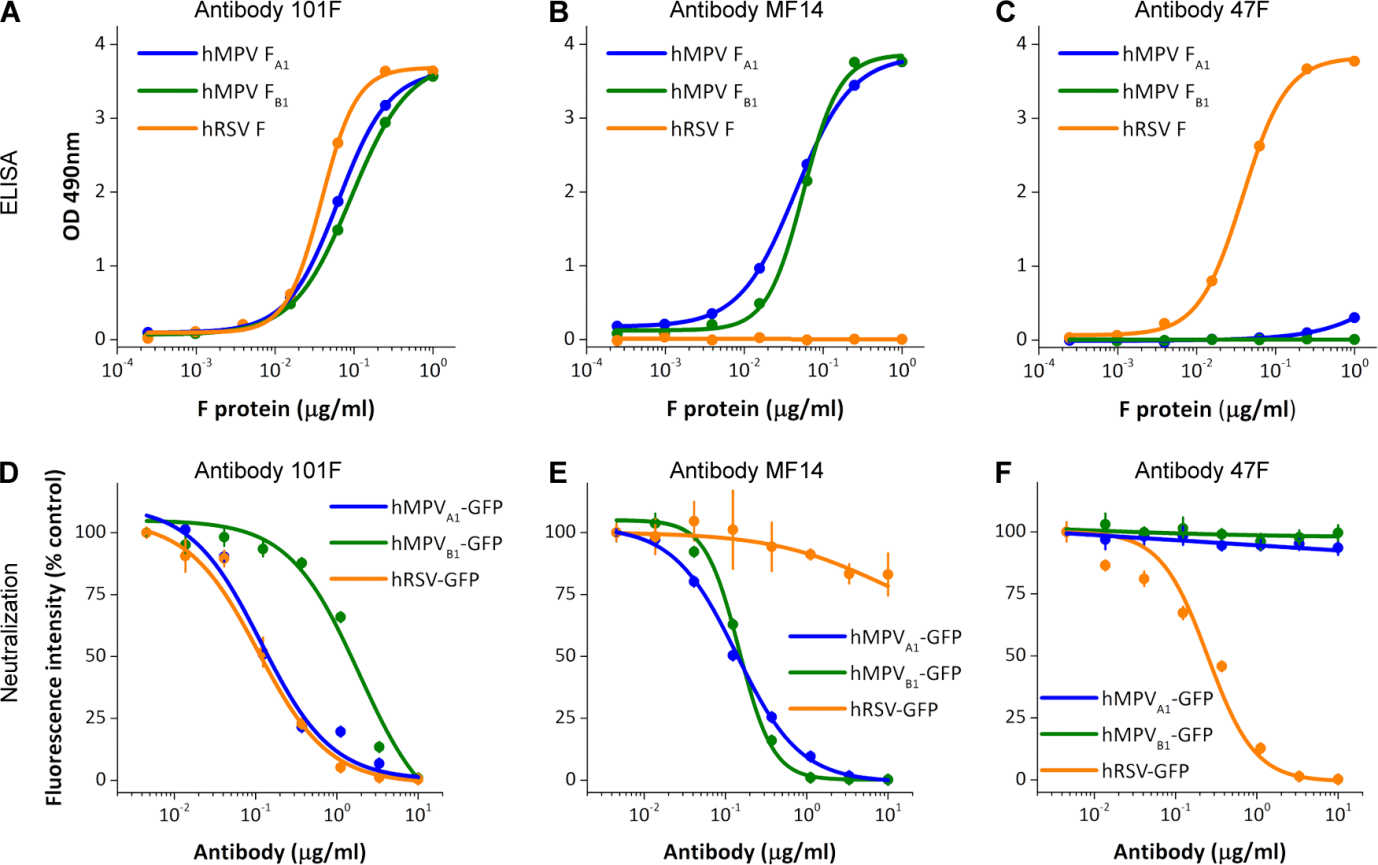
mAb 101F ELISA binding and neutralization. **A**, **B** and **C**) ELISA binding of mAb 101F, the hMPV-specific mAb MF14, and the hRSV-specific mAb 47F to the indicated postfusion proteins. **D**, **E** and **F**) Neutralization of the noted viruses with the three mAbs.

Binding of mAb 101F to soluble hMPV and hRSV postfusion F proteins was also tested by ELISA (Fig 5). The binding curves of 101F to hMPV F proteins from A1 or B1 lineages (Fig 5A) were similar to the binding curves of mAb MF14 (Fig 5B), which is a murine mAb specific for hMPV F (Fig 5B). In contrast, mAb 47F, specific for hRSV F [63], bound to the postfusion hRSV F protein as efficiently as 101F, but failed to bind significantly to the hMPV F proteins (Fig 5C).

The antibodies used in the ELISA tests were also used in microneutralization assays with the same viruses from which the F proteins originated (Fig 5). mAb MF14 neutralized the two hMPV strains with similar efficiency, and mAb 101F also neutralized those two viruses, although it was slightly less efficient than MF14 in neutralization of the NL/1/99 strain (B1 sublineage, Fig 5D and 5E). In agreement with the ELISA results, 47F failed to neutralize hMPV, but neutralized hRSV almost as efficiently as 101F (Fig 5F). MF14 did not neutralize hRSV infectivity, consistent with its lack of binding as determined by ELISA.

Since neutralization of hRSV by 101F is likely due to its binding to prefusion F [64], the binding of 101F to prefusion hMPV F was tested by an indirect method, since a prefusion-stabilized form of hMPV F is still unavailable. Thus, Vero-118 cells were infected with hMPV_A1_-GFP for 36 hours and then stained with 101F and control mAbs (S3 Fig). Antibody binding was revealed by confocal microscopy (left panels) or flow cytometry (right panels). The staining intensities of mAbs MF14 (a neutralizing antibody specific for hMPV F, Fig 5) and 101F were equivalent to that of mAb MPE8, which preferentially binds to the prefusion conformation [38]. Staining with mAb MF1, specific for the 6HB motif of postfusion F, was 5–10 times lower (see mean fluorescence intensity values in the flow cytometry panels). Although these data suggest that 101F is able to bind prefusion hMPV F and that this is the predominant conformation at the time point of S3 Fig, the relative affinities of the antibodies for each conformation are unknown. Therefore, a similar flow cytometry experiment was performed with or without heating of the cells at 50°C for 10 minutes, which should be sufficient to convert the majority of hMPV F from the pre- to postfusion conformation. As expected, the binding of MF1 was enhanced with the heat treatment (S4 Fig), whereas the binding of antibodies MF14, 101F and MPE8 were largely unchanged or modestly decreased. Collectively, these data, along with the SPR (Fig 4) and neutralization (Fig 5) results, indicate that 101F binds to both the pre- and postfusion forms of hMPV F.

**Fig 6:**
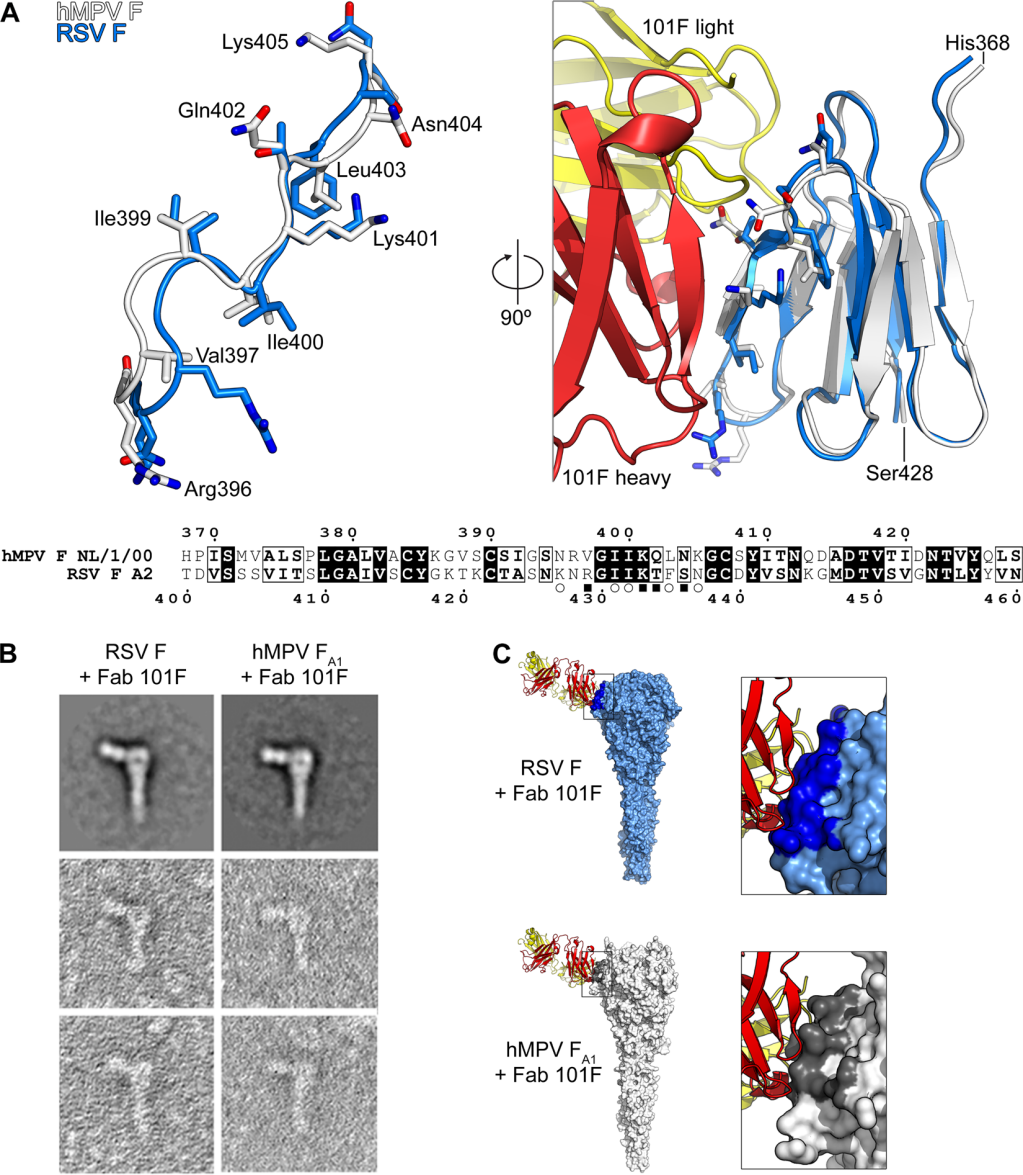
Structural basis of 101F cross-reactivity. **A**) *Left*: Superposition of the linear 101F epitope derived from the hMPV (white) and hRSV (blue) postfusion F proteins. Side-chains are shown as sticks, with oxygen atoms colored red and nitrogen atoms colored blue. hMPV F residues are labeled and numbered. *Right*: Model of mAb 101F bound to antigenic site IV derived from the hMPV and hRSV postfusion F proteins. 101F heavy chain is colored red and light chain is yellow. *Bottom*: Sequence alignment of the antigenic site IV domain from hMPV and hRSV F. Identical residues have white text with black backgrounds, whereas residues that are similar are in bold and have a black border. Open circles denote residues with >10 Å^2^ buried surface area and filled rectangles denote residues whose side-chains form hydrogen bonds with 101F in the hRSV F peptide-bound crystal structure (PDBID: 3O45). **B**) Negatively stained electron micrographs of 101F Fab bound to postfusion hRSV F and hMPV F. The top two panels are 2D averages whereas the other panels are examples of individual negatively stained F–Fab complexes. **C**) Models of a single 101F Fab in complex with postfusion F trimers of hRSV and hMPV. Molecular surfaces of the trimers are shown, and residues within 5.5 Å of 101F atoms are darker.

To provide a structural basis for 101F cross-reactivity, we compared its epitope in both the hRSV and hMPV postfusion F structures (Fig 6). Based on our previous crystal structures of 101F in complex with hRSV F-derived peptides, we defined the minimal epitope as hRSV F residues 427–437, which corresponds to hMPV F residues 395–405 [40]. In the postfusion F structures, these 11 residues are in a similar conformation, with an rmsd of 2.38 Å for the Cα atoms (Fig 6A). Importantly, all four residues in the center of the epitope are the same in both viruses, including the critical Lys433 residue (hRSV numbering; 401 in hMPV) that is altered in a 101F-escape variant (K433T) [39]. Substitutions at other residues within the epitope likely account for the decreased affinity of 101F for hMPV F. As expected based on the structural similarity, visualization of the complex by negative-stain EM revealed a binding mode essentially identical to that observed for the complex of 101F bound to postfusion hRSV F (Fig 6B).The images agreed well with models of 101F bound to hRSV and hMPV postfusion F proteins, which were based on superpositions of the peptide-bound 101F complex and the postfusion F proteins (Fig 6C).

### Immunogenicity of the purified soluble postfusion hMPV F glycoprotein

Postfusion forms of hRSV F, either as soluble ectodomain [28] or full-length protein aggregated in rosettes [65], have shown their potential to induce neutralizing antibodies in cotton rats and to protect them against a virus challenge. Similarly, soluble forms of hMPV F have been used to immunize cotton rats [66], Syrian golden hamsters [67], BALB/c mice [68] and macaques [69], demonstrating the capacity of hMPV F to induce neutralizing antibodies and protection.

However, the soluble forms of hMPV F used in previous immunizations were not stabilized and purified as described above and probably represented a heterogeneous mixture of different conformers. Hence, it was pertinent to test the immunogenic potential of the well-characterized and crystallized hMPV postfusion F protein from the NL/1/00 strain (A1 sublineage) in BALB/c mice. Mice were also immunized with the equivalent F protein from the NL/1/99 strain (B1 sublineage) as well as an equivalent soluble form of postfusion hRSV F. Mice were inoculated after mixing the proteins with CpG as adjuvant. A single dose rather than multiple doses was used to discriminate better the specificity of the antibodies induced by each protein.

**Fig 7:**
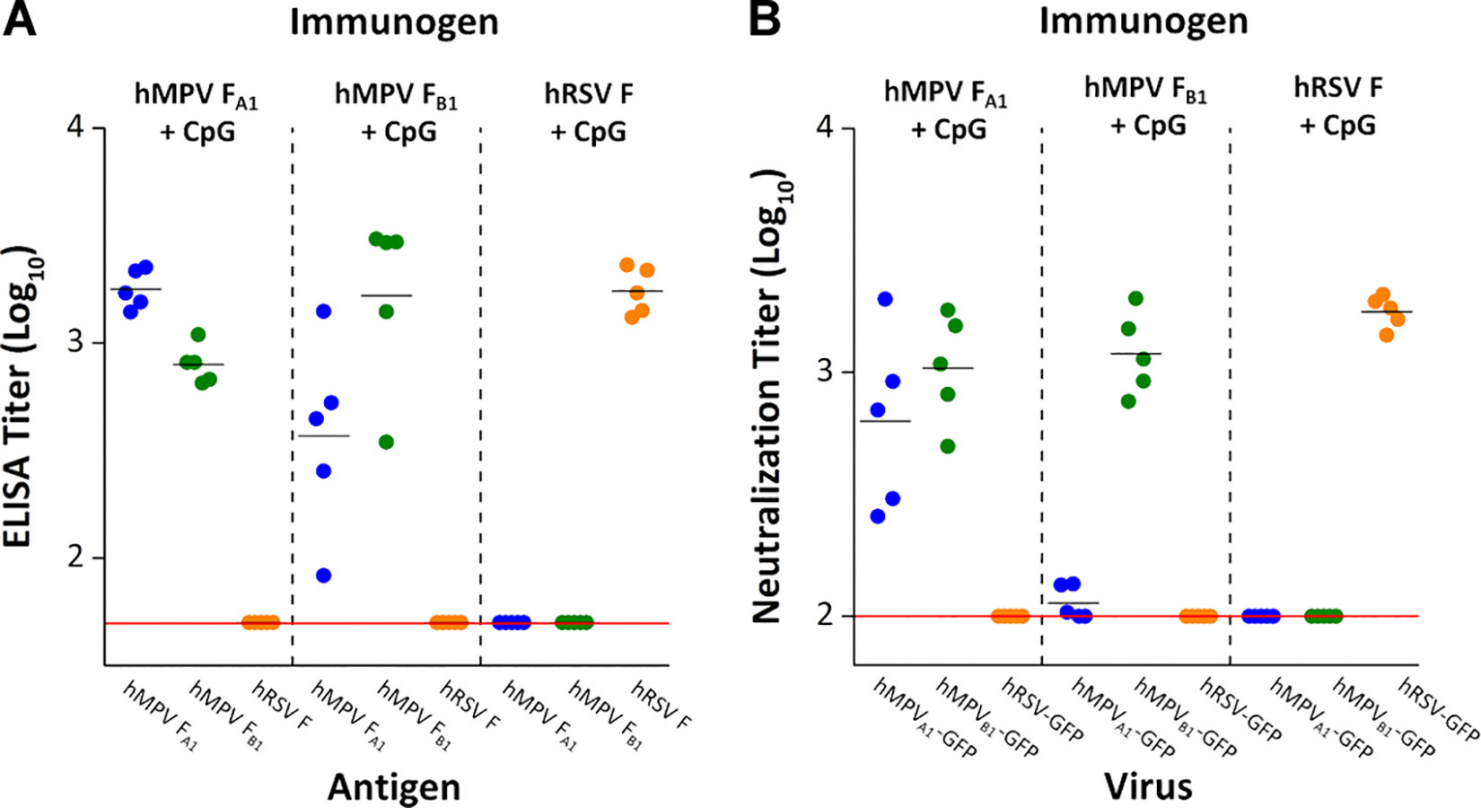
Binding and neutralization of sera from mice immunized with postfusion F proteins. Soluble hMPV F postfusion trimers derived from NL/1/00 (A1 sublineage) or NL/1/99 (B1 sublineage) viruses and the equivalent hRSV F postfusion trimer were used to immunize BALB/c mice. Sera were collected 4 weeks after immunization and tested in (**A**) ELISA and (**B**) neutralization assays. ELISA titers refer to serum dilution that yielded 50% of the maximal (saturating) value and neutralization titers refer to dilution that inhibited 50% fluorescence intensity, 48 hours after infection. Mean ELISA and neutralization titers for each group are shown by horizontal bars. Horizontal red lines in each panel indicate detection limits.

The sera of mice inoculated with the hMPV F proteins showed high levels of antibodies binding to homologous and heterologous hMPV postfusion F proteins, but had no binding activity to the soluble postfusion hRSV F (Fig 7A). In addition, the binding titers were significantly higher for the homologous hMPV F protein versus the heterologous hMPV F protein, particularly in sera of mice inoculated with NL/1/99 (B1 sublineage).

Mouse sera were also tested in a microneutralization assay (Fig 7B). As with ELISA, the sera of mice inoculated with the hMPV F protein from the NL/1/00 strain (A1 sublineage) neutralized both the homologous and the heterologous hMPV strains with similar efficiencies. In contrast, the sera of mice inoculated with the F protein from the NL/1/99 strain (B1 sublineage) had significantly higher neutralizing titers against the homologous strain than against the heterologous strain. Of note, the sera of mice inoculated with either of the two hMPV F proteins failed to neutralize hRSV (A2 strain). These data indicate that the postfusion conformation of the hMPV F protein is highly immunogenic, but fails to elicit hRSV-neutralizing activity despite a high degree of structural similarity with the postfusion hRSV F protein (Fig 3) and cross-reactivity of antibodies 101F (this article), 54G10 [37] and MPE8 [38]. Reciprocally, the sera of mice inoculated with a soluble form of postfusion hRSV F neutralized hRSV but failed to neutralize either of the two hMPV strains (Fig 7B).

## Discussion

Data accumulated during the last 10–15 years have demonstrated extensive similarities between the clinical manifestations and epidemiology of hMPV and hRSV [2], and the two viruses share many steps of their respective life cycles. However, there are also important differences in gene order and number of genes encoded in their genomes [10] as well as in individual gene products. One of these differences resides in the protease maturation of their respective F glycoproteins. Whereas the hMPV F precursor is cleaved only once by trypsin-like proteases, the hRSV F precursor is cleaved at two distinct furin sites separated by 27 amino acids [70], which is unique among paramyxoviruses. It is still unresolved whether or not this difference has an impact on structural and functional properties of their respective F glycoproteins, but it has been shown that insertion of the two cleavage sites of hRSV F into Sendai virus F protein changed the hemagglutinin-neuraminidase (HN) dependence of Sendai F for activation [71] and altered Sendai virus thermostability [72].

It is conceivable therefore that hMPV F may be less stable than other fusion proteins and hence requires the genetic manipulations shown in Fig 1A to fold into a stable postfusion trimer. Indeed, whereas the ectodomain of hMPV F without an additional trimerization domain (Fig 1A, construct 1) is found mainly as a monomer in the supernatant of cultured cells, the ectodomains of uncleaved hPIV3 F [31], NDV F [30] and hRSV F [56] or cleaved hRSV F [28,29] fold spontaneously as stable trimers. Stabilization of the hMPV F postfusion trimer was dependent on cleavage of the protein precursor. As seen in Fig 1D, cleavage increased thermostability of several epitopes in postfusion hMPV F, an effect that had not been previously observed for the homologous hRSV F [57]. Given the high similarity between postfusion hMPV and hRSV F structures (Fig 3A), the source of their apparent differences in stability remains to be determined.

As mentioned in the Introduction, cross-neutralization of hMPV and hRSV has been observed with certain anti-F mAbs. Particularly, 54G10, a human-derived mAb selected for binding to hMPV F, was shown to neutralize hRSV *in vitro* and to confer passive protection of mice against a hRSV challenge [37]. 54G10 selected hMPV escape mutants with an alteration (V397G) at a residue located in the corresponding region of the hRSV F antigenic site IV (Fig 6A). In a reciprocal manner, we have shown here that 101F, a murine mAb selected for binding to hRSV F, is capable of cross-binding to hMPV F and cross-neutralizing hMPV infectivity. Fig 6 provides an explanation for 101F cross-reactivity with the two F proteins since its binding site is fairly conserved in both hMPV F and hRSV F structures. The small differences in the structure of site IV between the two proteins may account for the noted affinity differences (Fig 4), which nevertheless are not linearly translated into neutralization potency (Fig 5).

It is worth mentioning that 101F also binds to hRSV F in the prefusion conformation [64], and this is likely responsible for its neutralizing activity. In a similar manner, the neutralization of hMPV by 101F probably requires binding to its prefusion F. This hypothesis is supported by the results shown in S3 and S4 Figs, which indicate binding of 101F to prefusion F expressed at the surface of hMPV-infected cells.

Despite the noted cross-reactivity with certain mAbs, neither significant cross-reactivity nor cross-neutralization were evident with the polyclonal antibodies induced in mice after inoculation with purified postfusion hMPV F or hRSV F. It should be noted that the cross-reactivity of mAb 101F with hMPV and hRSV F was revealed only after testing more than twelve different mAbs raised against hRSV F, including seven that competed with 101F for antigen binding. Although the structures of postfusion hMPV F and hRSV F are similar (Fig 3A), there is limited overall sequence identity (33–35%) [10]. Since antigen binding is dictated mainly by interactions of specific amino acid side-chains in the antibody with those in the antigen, it is likely that sequence changes in F, not reflected in the overall structure, account for the lack of polyclonal serum cross-reactivity and the scarce cross-reactivity of mAbs between postfusion hMPV F and hRSV F. An interim conclusion might be that antibodies that cross-neutralize hMPV and hRSV probably represent a minority of the global repertoire of specificities present in a polyclonal response, which is not just a pool of mAbs. Nevertheless, the sporadic isolation of cross-neutralizing mAbs that inhibit hMPV and hRSV infectivity opens the possibility of designing modified forms of the *Pneumovirinae* F capable of inducing highly cross-reactive and cross-protective antibody responses, as recently shown for the influenza virus hemagglutinin [73].

## Supporting Information

S1 Fig Effect of trypsin on hMPV F aggregation. **A**) Three micrograms of the purified hMPV F protein shown in Fig 1, construct 2, were treated with the indicated units of trypsin-agarose (Sigma) for 1 hour at 37°C before being loaded for SDS-PAGE and staining with Coomassie brilliant blue. Note cleavage of the F0 band and the emergence of the F1 band (and another low-molecular-weight band corresponding to spurious cleavage) with increasing amount of trypsin. **B**) An aliquot of the sample from panel A treated with 0.5 units of trypsin-agarose was observed by electron microscopy. Note the F molecules aggregated in rosettes, in comparison with the same protein (construct 2) before treatment, Fig 1C, panel 2. Scale bar: 50 nm.
(TIF)

S2 Fig Electron microscopy of postfusion hMPV F_B1_. Negatively stained electron micrograph of the hMPV F protein corresponding to the same construct shown in Fig 1C, panel 3, but derived from the NL/1/99 strain (B1 sublineage). Scale bar: 50 nm.
(TIF)

S3 Fig Surface labelling of hMPV-infected Vero-118 cells. Cells were infected with hMPV_A1_-GFP virus for 36 hours (green color corresponds to infected cells), and then stained with the mAbs shown on the left. Primary antibodies were detected with streptavidin-RPE secondary antibodies (red color), and the cells were observed by confocal microscopy (left panels) and flow cytometry (right panels). Numbers in the Q2 sector of each fluorogram indicate percentage of doubly stained cells and mean fluorescence intensity of antibody labelling (PE-A mean).
(TIF)

S4 Fig Effect of heating on surface labelling of hMPV-infected Vero-118 cells. Vero-118 cells were grown and infected with hMPV_A1_ virus, as indicated in the legend of S3 Fig. Twenty-four hours after infection, the cultures were either left at 37°C (solid pink histogram) or shifted to 50°C for 10 minutes (empty red histogram). Then, medium was removed and the cells were processed for flow cytometry as in S3 Fig with the antibodies indicated in each panel. The mock-infected control is shown as a solid grey histogram. The mean fluorescence intensity (PE) and the percentage of cells in the P1 population are indicated in each panel.
(TIF)

## References

[1] van den Hoogen BG, de Jong JC, Groen J, Kuiken T, de Groot R, Fouchier RA, Osterhaus AD (2001) A newly discovered human pneumovirus isolated from young children with respiratory tract disease. Nat Med 7: 719–724. . 11385510 10.1038/89098 PMC7095854

[2] Schildgen V, van den Hoogen B, Fouchier R, Tripp RA, Alvarez R, Manoha C, Williams J, Schildgen O (2011) Human Metapneumovirus: lessons learned over the first decade. Clin Microbiol Rev 24: 734–754. 10.1128/CMR.00015-11 21976607 PMC3194831

[3] van den Hoogen BG, Herfst S, Sprong L, Cane PA, Forleo-Neto E, de Swart RL, Osterhaus AD, Fouchier RA (2004) Antigenic and genetic variability of human metapneumoviruses. Emerg Infect Dis 10: 658–666. . 15200856 10.3201/eid1004.030393 PMC3323073

[4] Shi T, McLean K, Campbell H, Nair H (2015) Aetiological role of common respiratory viruses in acute lower respiratory infections in children under five years: A systematic review and meta-analysis. J Glob Health 5: 010408 10.7189/jogh.05.010408 26445672 PMC4593292

[5] Falsey AR, McElhaney JE, Beran J, van Essen GA, Duval X, Esen M, Galtier F, Gervais P, Hwang SJ, Kremsner P, Launay O, Leroux-Roels G, McNeil SA, Nowakowski A, Richardus JH, Ruiz-Palacios G, St RS, Devaster JM, Oostvogels L, Durviaux S, Taylor S (2014) Respiratory syncytial virus and other respiratory viral infections in older adults with moderate to severe influenza-like illness. J Infect Dis 209: 1873–1881. 10.1093/infdis/jit839 24482398 PMC4038137

[6] Boivin G, De Serres G, Hamelin ME, Cote S, Argouin M, Tremblay G, Maranda-Aubut R, Sauvageau C, Ouakki M, Boulianne N, Couture C (2007) An outbreak of severe respiratory tract infection due to human metapneumovirus in a long-term care facility. Clin Infect Dis 44: 1152–1158. . 17407031 10.1086/513204

[7] Panda S, Mohakud NK, Pena L, Kumar S (2014) Human metapneumovirus: review of an important respiratory pathogen. Int J Infect Dis 25: 45–52. 10.1016/j.ijid.2014.03.1394 24841931 PMC7110553

[8] Godet C, Le GJ, Beby-Defaux A, Robin M, Raffoux E, Arnulf B, Roblot F, Frat JP, Maillard N, Tazi A, Bergeron A (2014) Human metapneumovirus pneumonia in patients with hematological malignancies. J Clin Virol 61: 593–596. 10.1016/j.jcv.2014.08.019 25440914 PMC7173302

[9] Bao X, Kolli D, Liu T, Shan Y, Garofalo RP, Casola A (2008) Human metapneumovirus small hydrophobic protein inhibits NF-kappaB transcriptional activity. J Virol 82: 8224–8229. 10.1128/JVI.02584-07 18550666 PMC2519579

[10] van den Hoogen BG, Bestebroer TM, Osterhaus AD, Fouchier RA (2002) Analysis of the genomic sequence of a human metapneumovirus. Virology 295: 119–132. . 12033771 10.1006/viro.2001.1355

[11] Thammawat S, Sadlon TA, Hallsworth PG, Gordon DL (2008) Role of cellular glycosaminoglycans and charged regions of viral G protein in human metapneumovirus infection. J Virol 82: 11767–11774. 10.1128/JVI.01208-08 18786997 PMC2583676

[12] Cox RG, Livesay SB, Johnson M, Ohi MD, Williams JV (2012) The human metapneumovirus fusion protein mediates entry via an interaction with RGD-binding integrins. J Virol 86: 12148–12160. 10.1128/JVI.01133-12 22933271 PMC3486500

[13] Melero JA, Mas V (2015) The Pneumovirinae fusion (F) protein: A common target for vaccines and antivirals. Virus Res 209: 128–135. 10.1016/j.virusres.2015.02.024 25738581

[14] Biacchesi S, Skiadopoulos MH, Yang L, Lamirande EW, Tran KC, Murphy BR, Collins PL, Buchholz UJ (2004) Recombinant human Metapneumovirus lacking the small hydrophobic SH and/or attachment G glycoprotein: deletion of G yields a promising vaccine candidate. J Virol 78: 12877–12887. . 15542640 10.1128/JVI.78.23.12877-12887.2004 PMC525014

[15] Chang A, Masante C, Buchholz UJ, Dutch RE (2012) Human metapneumovirus (HMPV) binding and infection are mediated by interactions between the HMPV fusion protein and heparan sulfate. J Virol 86: 3230–3243. 10.1128/JVI.06706-11 22238303 PMC3302303

[16] Cseke G, Maginnis MS, Cox RG, Tollefson SJ, Podsiad AB, Wright DW, Dermody TS, Williams JV (2009) Integrin alphavbeta1 promotes infection by human metapneumovirus. Proc Natl Acad Sci U S A 106: 1566–1571. 10.1073/pnas.0801433106 19164533 PMC2629439

[17] Wei Y, Zhang Y, Cai H, Mirza AM, Iorio RM, Peeples ME, Niewiesk S, Li J (2014) Roles of the putative integrin-binding motif of the human metapneumovirus fusion (f) protein in cell-cell fusion, viral infectivity, and pathogenesis. J Virol 88: 4338–4352. 10.1128/JVI.03491-13 24478423 PMC3993731

[18] Cox RG, Mainou BA, Johnson M, Hastings AK, Schuster JE, Dermody TS, Williams JV (2015) Human Metapneumovirus Is Capable of Entering Cells by Fusion with Endosomal Membranes. PLoS Pathog 11: e1005303 10.1371/journal.ppat.1005303 26629703 PMC4667933

[19] Schowalter RM, Smith SE, Dutch RE (2006) Characterization of human metapneumovirus F protein-promoted membrane fusion: critical roles for proteolytic processing and low pH. J Virol 80: 10931–10941. . 16971452 10.1128/JVI.01287-06 PMC1642150

[20] Herfst S, Mas V, Ver LS, Wierda RJ, Osterhaus AD, Fouchier RA, Melero JA (2008) Low-pH-induced membrane fusion mediated by human metapneumovirus F protein is a rare, strain-dependent phenomenon. J Virol 82: 8891–8895. 10.1128/JVI.00472-08 18596097 PMC2519679

[21] Le NC, Hillyer P, Brock LG, Winter CC, Rabin RL, Collins PL, Buchholz UJ (2014) Human metapneumovirus SH and G glycoproteins inhibit macropinocytosis-mediated entry into human dendritic cells and reduce CD4+ T cell activation. J Virol 88: 6453–6469. 10.1128/JVI.03261-13 24672038 PMC4093882

[22] Schickli JH, Kaur J, Ulbrandt N, Spaete RR, Tang RS (2005) An S101P substitution in the putative cleavage motif of the human metapneumovirus fusion protein is a major determinant for trypsin-independent growth in vero cells and does not alter tissue tropism in hamsters. J Virol 79: 10678–10689. .16051860

[23] Shirogane Y, Takeda M, Iwasaki M, Ishiguro N, Takeuchi H, Nakatsu Y, Tahara M, Kikuta H, Yanagi Y (2008) Efficient multiplication of human metapneumovirus in Vero cells expressing the transmembrane serine protease TMPRSS2. J Virol 82: 8942–8946. 10.1128/JVI.00676-08 18562527 PMC2519639

[24] McLellan JS, Chen M, Joyce MG, Sastry M, Stewart-Jones GB, Yang Y, Zhang B, Chen L, Srivatsan S, Zheng A, Zhou T, Graepel KW, Kumar A, Moin S, Boyington JC, Chuang GY, Soto C, Baxa U, Bakker AQ, Spits H, Beaumont T, Zheng Z, Xia N, Ko SY, Todd JP, Rao S, Graham BS, Kwong PD (2013) Structure-based design of a fusion glycoprotein vaccine for respiratory syncytial virus. Science 342: 592–598. 10.1126/science.1243283 24179220 PMC4461862

[25] McLellan JS, Chen M, Leung S, Graepel KW, Du X, Yang Y, Zhou T, Baxa U, Yasuda E, Beaumont T, Kumar A, Modjarrad K, Zheng Z, Zhao M, Xia N, Kwong PD, Graham BS (2013) Structure of RSV fusion glycoprotein trimer bound to a prefusion-specific neutralizing antibody. Science 340: 1113–1117. 10.1126/science.1234914 23618766 PMC4459498

[26] Welch BD, Liu Y, Kors CA, Leser GP, Jardetzky TS, Lamb RA (2012) Structure of the cleavage-activated prefusion form of the parainfluenza virus 5 fusion protein. Proc Natl Acad Sci U S A 109: 16672–16677. 10.1073/pnas.1213802109 23012473 PMC3478641

[27] Yin HS, Wen X, Paterson RG, Lamb RA, Jardetzky TS (2006) Structure of the parainfluenza virus 5 F protein in its metastable, prefusion conformation. Nature 439: 38–44. . 16397490 10.1038/nature04322 PMC7095149

[28] Swanson KA, Settembre EC, Shaw CA, Dey AK, Rappuoli R, Mandl CW, Dormitzer PR, Carfi A (2011) Structural basis for immunization with postfusion respiratory syncytial virus fusion F glycoprotein (RSV F) to elicit high neutralizing antibody titers. Proc Natl Acad Sci U S A 108: 9619–9624. 10.1073/pnas.1106536108 21586636 PMC3111287

[29] McLellan JS, Yang Y, Graham BS, Kwong PD (2011) Structure of respiratory syncytial virus fusion glycoprotein in the postfusion conformation reveals preservation of neutralizing epitopes. J Virol 85: 7788–7796. 10.1128/JVI.00555-11 21613394 PMC3147929

[30] Swanson K, Wen X, Leser GP, Paterson RG, Lamb RA, Jardetzky TS (2010) Structure of the Newcastle disease virus F protein in the post-fusion conformation. Virology 402: 372–379. 10.1016/j.virol.2010.03.050 20439109 PMC2877518

[31] Yin HS, Paterson RG, Wen X, Lamb RA, Jardetzky TS (2005) Structure of the uncleaved ectodomain of the paramyxovirus (hPIV3) fusion protein. Proc Natl Acad Sci U S A 102: 9288–9293. . 15964978 10.1073/pnas.0503989102 PMC1151655

[32] Jardetzky TS, Lamb RA (2014) Activation of Paramyxovirus Membrane Fusion and Virus Entry. Curr Opin Virol 0: 24–33. . 24530984 10.1016/j.coviro.2014.01.005 PMC4028362

[33] Wen SC, Williams JV (2015) New Approaches for Immunization and Therapy against Human Metapneumovirus. Clin Vaccine Immunol 22: 858–866. 10.1128/CVI.00230-15 26063237 PMC4519717

[34] Skiadopoulos MH, Biacchesi S, Buchholz UJ, Amaro-Carambot E, Surman SR, Collins PL, Murphy BR (2006) Individual contributions of the human metapneumovirus F, G, and SH surface glycoproteins to the induction of neutralizing antibodies and protective immunity. Virology 345: 492–501. . 16300813 10.1016/j.virol.2005.10.016

[35] Ryder AB, Tollefson SJ, Podsiad AB, Johnson JE, Williams JV (2010) Soluble recombinant human metapneumovirus G protein is immunogenic but not protective. Vaccine 28: 4145–4152. 10.1016/j.vaccine.2010.04.007 20417260 PMC2894472

[36] Ulbrandt ND, Ji H, Patel NK, Barnes AS, Wilson S, Kiener PA, Suzich J, McCarthy MP (2008) Identification of antibody neutralization epitopes on the fusion protein of human metapneumovirus. J Gen Virol 89: 3113–3118. 10.1099/vir.0.2008/005199-0 19008400 PMC2885031

[37] Schuster JE, Cox RG, Hastings AK, Boyd KL, Wadia J, Chen Z, Burton DR, Williamson RA, Williams JV (2014) A Broadly Neutralizing Human Monoclonal Antibody Exhibits In Vivo Efficacy Against Both Human Metapneumovirus and Respiratory Syncytial Virus. J Infect Dis. . 24864121 10.1093/infdis/jiu307 PMC4342691

[38] Corti D, Bianchi S, Vanzetta F, Minola A, Perez L, Agatic G, Guarino B, Silacci C, Marcandalli J, Marsland BJ, Piralla A, Percivalle E, Sallusto F, Baldanti F, Lanzavecchia A (2013) Cross-neutralization of four paramyxoviruses by a human monoclonal antibody. Nature 501: 439–443. 10.1038/nature12442 23955151

[39] Wu SJ, Schmidt A, Beil EJ, Day ND, Branigan PJ, Liu C, Gutshall LL, Palomo C, Furze J, Taylor G, Melero JA, Tsui P, Del Vecchio AM, Kruszynski M (2007) Characterization of the epitope for anti-human respiratory syncytial virus F protein monoclonal antibody 101F using synthetic peptides and genetic approaches. J Gen Virol 88: 2719–2723. . 17872524 10.1099/vir.0.82753-0

[40] McLellan JS, Chen M, Chang JS, Yang Y, Kim A, Graham BS, Kwong PD (2010) Structure of a major antigenic site on the respiratory syncytial virus fusion glycoprotein in complex with neutralizing antibody 101F. J Virol 84: 12236–12244. 10.1128/JVI.01579-10 20881049 PMC2976384

[41] Mas V, Herfst S, Osterhaus AD, Fouchier RA, Melero JA (2011) Residues of the human metapneumovirus fusion (F) protein critical for its strain-related fusion phenotype: implications for the virus replication cycle. J Virol 85: 12650–12661. 10.1128/JVI.05485-11 21937649 PMC3209396

[42] Meier S, Guthe S, Kiefhaber T, Grzesiek S (2004) Foldon, the natural trimerization domain of T4 fibritin, dissociates into a monomeric A-state form containing a stable beta-hairpin: atomic details of trimer dissociation and local beta-hairpin stability from residual dipolar couplings. J Mol Biol 344: 1051–1069. . 15544812 10.1016/j.jmb.2004.09.079

[43] Blasco R, Moss B (1995) Selection of recombinant vaccinia viruses on the basis of plaque formation. Gene 158: 157–162. . 7607536 10.1016/0378-1119(95)00149-z

[44] Vey M, Schafer W, Berghofer S, Klenk HD, Garten W (1994) Maturation of the trans-Golgi network protease furin: compartmentalization of propeptide removal, substrate cleavage, and COOH-terminal truncation. J Cell Biol 127: 1829–1842. . 7806563 10.1083/jcb.127.6.1829 PMC2120303

[45] Marabini R, Masegosa IM, San Martin MC, Marco S, Fernandez JJ, de la Fraga LG, Vaquerizo C, Carazo JM (1996) Xmipp: An Image Processing Package for Electron Microscopy. J Struct Biol 116: 237–240. . 8812978 10.1006/jsbi.1996.0036

[46] Majeed S, Ofek G, Belachew A, Huang CC, Zhou T, Kwong PD (2003) Enhancing protein crystallization through precipitant synergy. Structure 11: 1061–1070. . 12962625 10.1016/s0969-2126(03)00185-0

[47] Battye TG, Kontogiannis L, Johnson O, Powell HR, Leslie AG (2011) iMOSFLM: a new graphical interface for diffraction-image processing with MOSFLM. Acta Crystallogr D Biol Crystallogr 67: 271–281. 10.1107/S0907444910048675 21460445 PMC3069742

[48] Evans PR, Murshudov GN (2013) How good are my data and what is the resolution? Acta Crystallogr D Biol Crystallogr 69: 1204–1214. 10.1107/S0907444913000061 23793146 PMC3689523

[49] McCoy AJ, Grosse-Kunstleve RW, Adams PD, Winn MD, Storoni LC, Read RJ (2007) Phaser crystallographic software. J Appl Crystallogr 40: 658–674. . 19461840 10.1107/S0021889807021206 PMC2483472

[50] Wen X, Krause JC, Leser GP, Cox RG, Lamb RA, Williams JV, Crowe JE Jr., Jardetzky TS (2012) Structure of the human metapneumovirus fusion protein with neutralizing antibody identifies a pneumovirus antigenic site. Nat Struct Mol Biol 19: 461–463. 10.1038/nsmb.2250 22388735 PMC3546531

[51] Emsley P, Lohkamp B, Scott WG, Cowtan K (2010) Features and development of Coot. Acta Crystallogr D Biol Crystallogr 66: 486–501. 10.1107/S0907444910007493 20383002 PMC2852313

[52] Adams PD, Afonine PV, Bunkoczi G, Chen VB, Davis IW, Echols N, Headd JJ, Hung LW, Kapral GJ, Grosse-Kunstleve RW, McCoy AJ, Moriarty NW, Oeffner R, Read RJ, Richardson DC, Richardson JS, Terwilliger TC, Zwart PH (2010) PHENIX: a comprehensive Python-based system for macromolecular structure solution. Acta Crystallogr D Biol Crystallogr 66: 213–221. 10.1107/S0907444909052925 20124702 PMC2815670

[53] Afonine PV, Moriarty NW, Mustyakimov M, Sobolev OV, Terwilliger TC, Turk D, Urzhumtsev A, Adams PD (2015) FEM: feature-enhanced map. Acta Crystallogr D Biol Crystallogr 71: 646–666. 10.1107/S1399004714028132 25760612 PMC4356370

[54] Rodriguez L, Olmedillas E, Mas V, Vazquez M, Cano O, Terron MC, Luque D, Palomo C, Melero JA (2015) Generation of monoclonal antibodies specific of the postfusion conformtion of the *Pneumovirinae* Fusion (F) protein. J Virol Methods 1–8. . 26275682 10.1016/j.jviromet.2015.08.002 PMC7119586

[55] Calder LJ, Gonzalez-Reyes L, Garcia-Barreno B, Wharton SA, Skehel JJ, Wiley DC, Melero JA (2000) Electron microscopy of the human respiratory syncytial virus fusion protein and complexes that it forms with monoclonal antibodies. Virology 271: 122–131. . 10814577 10.1006/viro.2000.0279

[56] Begona Ruiz-Arguello M, Gonzalez-Reyes L, Calder LJ, Palomo C, Martin D, Saiz MJ, Garcia-Barreno B, Skehel JJ, Melero JA (2002) Effect of proteolytic processing at two distinct sites on shape and aggregation of an anchorless fusion protein of human respiratory syncytial virus and fate of the intervening segment. Virology 298: 317–326. . 12127793 10.1006/viro.2002.1497

[57] Ruiz-Arguello MB, Martin D, Wharton SA, Calder LJ, Martin SR, Cano O, Calero M, Garcia-Barreno B, Skehel JJ, Melero JA (2004) Thermostability of the human respiratory syncytial virus fusion protein before and after activation: implications for the membrane-fusion mechanism. J Gen Virol 85: 3677–3687. . 15557241 10.1099/vir.0.80318-0

[58] Davis IW, Leaver-Fay A, Chen VB, Block JN, Kapral GJ, Wang X, Murray LW, Arendall WB III, Snoeyink J, Richardson JS, Richardson DC (2007) MolProbity: all-atom contacts and structure validation for proteins and nucleic acids. Nucleic Acids Res 35: W375–W383. . 17452350 10.1093/nar/gkm216 PMC1933162

[59] Chen VB, Arendall WB III, Headd JJ, Keedy DA, Immormino RM, Kapral GJ, Murray LW, Richardson JS, Richardson DC (2010) MolProbity: all-atom structure validation for macromolecular crystallography. Acta Crystallogr D Biol Crystallogr 66: 12–21. 10.1107/S0907444909042073 20057044 PMC2803126

[60] Swanson K, Wen X, Leser GP, Paterson RG, Lamb RA, Jardetzky TS (2010) Structure of the Newcastle disease virus F protein in the post-fusion conformation. Virology 402: 372–379. 10.1016/j.virol.2010.03.050 20439109 PMC2877518

[61] Lopez JA, Bustos R, Orvell C, Berois M, Arbiza J, Garcia-Barreno B, Melero JA (1998) Antigenic structure of human respiratory syncytial virus fusion glycoprotein. J Virol 72: 6922–6928. . 9658147 10.1128/jvi.72.8.6922-6928.1998 PMC109907

[62] Palomo C, Mas V, Vazquez M, Cano O, Luque D, Terron MC, Calder LJ, Melero JA (2014) Polyclonal and monoclonal antibodies specific for the six-helix bundle of the human respiratory syncytial virus fusion glycoprotein as probes of the protein post-fusion conformation. Virology 460-461C: 119–127. . 25010277 10.1016/j.virol.2014.05.001

[63] Garcia-Barreno B, Palomo C, Penas C, Delgado T, Perez-Brena P, Melero JA (1989) Marked differences in the antigenic structure of human respiratory syncytial virus F and G glycoproteins. J Virol 63: 925–932. . 2463385 10.1128/jvi.63.2.925-932.1989 PMC247767

[64] Magro M, Mas V, Chappell K, Vazquez M, Cano O, Luque D, Terron MC, Melero JA, Palomo C (2012) Neutralizing antibodies against the preactive form of respiratory syncytial virus fusion protein offer unique possibilities for clinical intervention. Proc Natl Acad Sci U S A 109: 3089–3094. 10.1073/pnas.1115941109 22323598 PMC3286924

[65] Smith G, Raghunandan R, Wu Y, Liu Y, Massare M, Nathan M, Zhou B, Lu H, Boddapati S, Li J, Flyer D, Glenn G (2012) Respiratory syncytial virus fusion glycoprotein expressed in insect cells form protein nanoparticles that induce protective immunity in cotton rats. PLoS One 7: e50852 10.1371/journal.pone.0050852 23226404 PMC3511306

[66] Cseke G, Wright DW, Tollefson SJ, Johnson JE, Crowe JE Jr., Williams JV (2007) Human metapneumovirus fusion protein vaccines that are immunogenic and protective in cotton rats. J Virol 81: 698–707. . 17050599 10.1128/JVI.00844-06 PMC1797435

[67] Herfst S, de Graaf M, Schrauwen EJ, Ulbrandt ND, Barnes AS, Senthil K, Osterhaus AD, Fouchier RA, van den Hoogen BG (2007) Immunization of Syrian golden hamsters with F subunit vaccine of human metapneumovirus induces protection against challenge with homologous or heterologous strains. J Gen Virol 88: 2702–2709. . 17872522 10.1099/vir.0.83084-0

[68] Aerts L, Rheaume C, Carbonneau J, Lavigne S, Couture C, Hamelin ME, Boivin G (2015) Adjuvant effect of the human metapneumovirus (HMPV) matrix protein in HMPV subunit vaccines. J Gen Virol 96: 767–774. 10.1099/vir.0.000031 25519171

[69] Herfst S, Schrauwen EJ, de GM, van AG, van den Hoogen BG, de Swart RL, Osterhaus AD, Fouchier RA (2008) Immunogenicity and efficacy of two candidate human metapneumovirus vaccines in cynomolgus macaques. Vaccine 26: 4224–4230. 10.1016/j.vaccine.2008.05.052 18585830

[70] Gonzalez-Reyes L, Ruiz-Arguello MB, Garcia-Barreno B, Calder L, Lopez JA, Albar JP, Skehel JJ, Wiley DC, Melero JA (2001) Cleavage of the human respiratory syncytial virus fusion protein at two distinct sites is required for activation of membrane fusion. Proc Natl Acad Sci U S A 98: 9859–9864. . 11493675 10.1073/pnas.151098198 PMC55543

[71] Rawling J, Garcia-Barreno B, Melero JA (2008) Insertion of the two cleavage sites of the respiratory syncytial virus fusion protein in Sendai virus fusion protein leads to enhanced cell-cell fusion and a decreased dependency on the HN attachment protein for activity. J Virol 82: 5986–5998. 10.1128/JVI.00078-08 18385247 PMC2395136

[72] Rawling J, Cano O, Garcin D, Kolakofsky D, Melero JA (2011) Recombinant sendai viruses expressing fusion proteins with two furin cleavage sites mimic the syncytial and receptor-independent infection properties of respiratory syncytial virus. J Virol 85: 2771–2780. 10.1128/JVI.02065-10 21228237 PMC3067931

[73] Yassine HM, Boyington JC, McTamney PM, Wei CJ, Kanekiyo M, Kong WP, Gallagher JR, Wang L, Zhang Y, Joyce MG, Lingwood D, Moin SM, Andersen H, Okuno Y, Rao SS, Harris AK, Kwong PD, Mascola JR, Nabel GJ, Graham BS (2015) Hemagglutinin-stem nanoparticles generate heterosubtypic influenza protection. Nat Med 21: 1065–1070. 10.1038/nm.3927 26301691

