# Supplementary figures and images for: Engineering, Structure and Immunogenicity of the Human Metapneumovirus F Protein in the Postfusion Conformation
Source: PLoS Pathog. 2016 Sep 9;12(9):e1005859. doi: 10.1371/journal.ppat.1005859 (PMC5017722; doi:10.1371/journal.ppat.1005859)

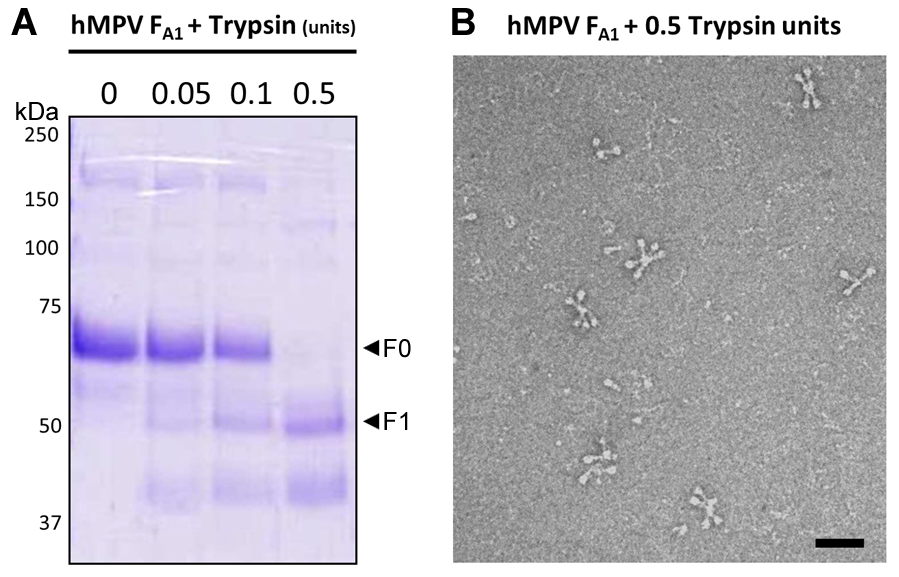

Supplement: S1 Fig — A) Three micrograms of the purified hMPV F protein shown in Fig 1, construct 2, were treated with the indicated units of trypsin-agarose (Sigma) for 1 hour at 37°C before being loaded for SDS-PAGE and staining with Coomassie brilliant blue. Note cleavage of the F0 band and the emergence of the F1 band (and another low-molecular-weight band corresponding to spurious cleavage) with increasing amount of trypsin. B) An aliquot of the sample from panel A treated with 0.5 units of trypsin-agarose was observed by electron microscopy. Note the F molecules aggregated in rosettes, in comparison with the same protein (construct 2) before treatment, Fig 1C, panel 2. Scale bar: 50 nm. (TIF) [file ppat.1005859.s001.tif]

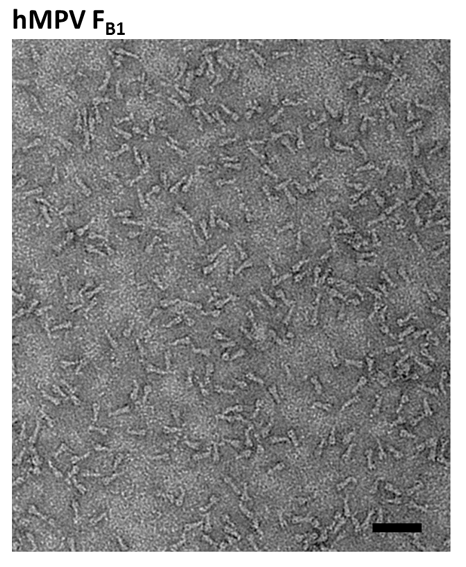

Supplement: S2 Fig — Negatively stained electron micrograph of the hMPV F protein corresponding to the same construct shown in Fig 1C, panel 3, but derived from the NL/1/99 strain (B1 sublineage). Scale bar: 50 nm. (TIF) [file ppat.1005859.s002.tif]

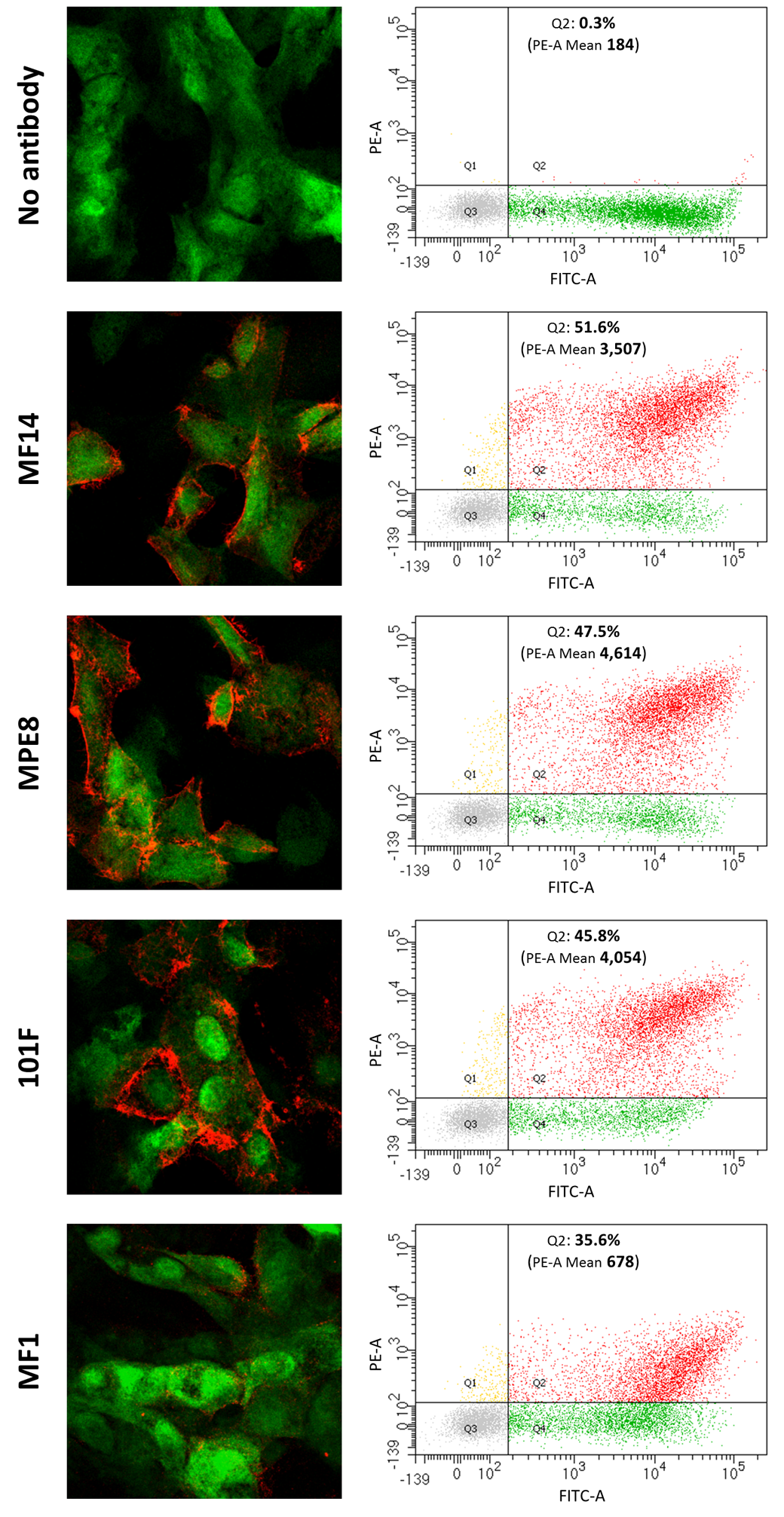

Supplement: S3 Fig — Cells were infected with hMPVA1-GFP virus for 36 hours (green color corresponds to infected cells), and then stained with the mAbs shown on the left. Primary antibodies were detected with streptavidin-RPE secondary antibodies (red color), and the cells were observed by confocal microscopy (left panels) and flow cytometry (right panels). Numbers in the Q2 sector of each fluorogram indicate percentage of doubly stained cells and mean fluorescence intensity of antibody labelling (PE-A mean). (TIF) [file ppat.1005859.s003.tif]

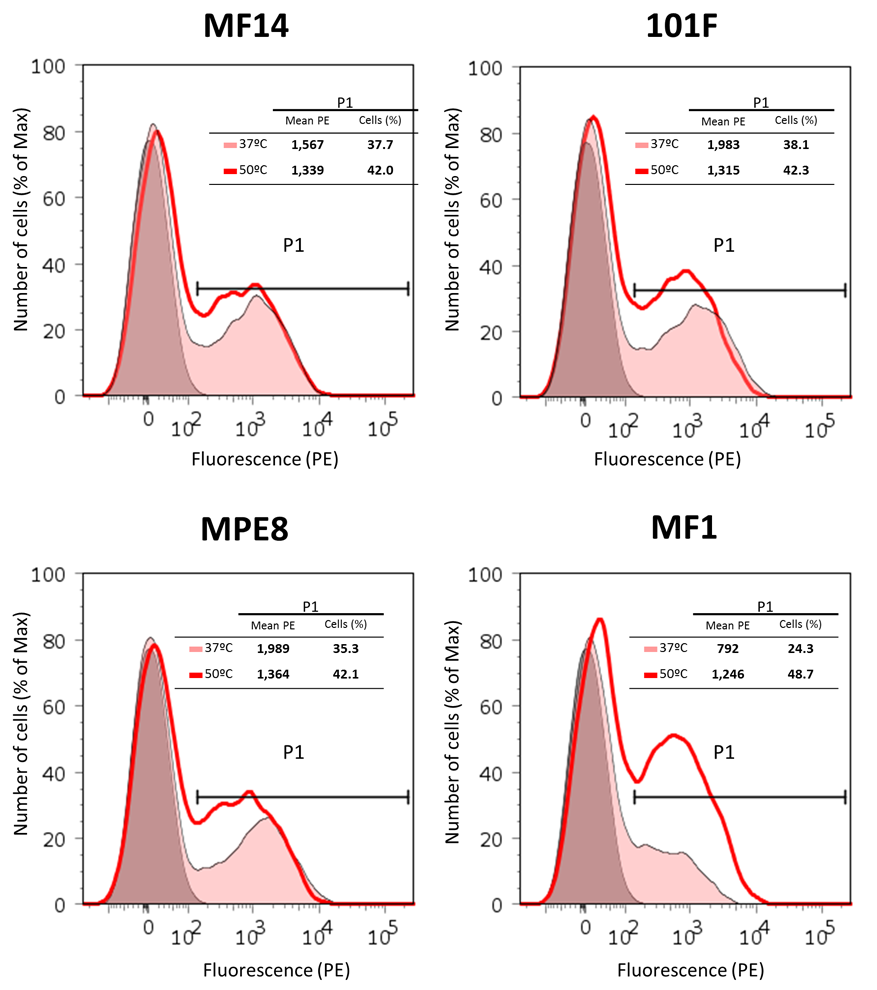

Supplement: S4 Fig — Vero-118 cells were grown and infected with hMPVA1 virus, as indicated in the legend of S3 Fig. Twenty-four hours after infection, the cultures were either left at 37°C (solid pink histogram) or shifted to 50°C for 10 minutes (empty red histogram). Then, medium was removed and the cells were processed for flow cytometry as in S3 Fig with the antibodies indicated in each panel. The mock-infected control is shown as a solid grey histogram. The mean fluorescence intensity (PE) and the percentage of cells in the P1 population are indicated in each panel. (TIF) [file ppat.1005859.s004.tif]
